# Enhancer-promoter interaction maps provide insights into skeletal muscle-related traits in pig genome

**DOI:** 10.1186/s12915-022-01322-2

**Published:** 2022-06-09

**Authors:** Jingjin Li, Yue Xiang, Lu Zhang, Xiaolong Qi, Zhuqing Zheng, Peng Zhou, Zhenshuang Tang, Yi Jin, Qiulin Zhao, Yuhua Fu, Yunxia Zhao, Xinyun Li, Liangliang Fu, Shuhong Zhao

**Affiliations:** 1 Key Laboratory of Agricultural Animal Genetics, Breeding and Reproduction of Ministry of Education & Key Lab of Swine Genetics and Breeding of Ministry of Agriculture and Rural Affairs, Huazhong Agricultural University, Wuhan, 430070 People’s Republic of China; 2 The Cooperative Innovation Center for Sustainable Pig Production, Huazhong Agricultural University, Wuhan, 430070 People’s Republic of China; 3 Hubei Hongshan Laboratory, 430070 Wuhan, People’s Republic of China

**Keywords:** H3K27ac BL-HiChIP, GRID-seq, GWAS, Skeletal muscle, Pigs

## Abstract

**Background:**

Gene expression programs are intimately linked to the interplay of active *cis* regulatory elements mediated by chromatin contacts and associated RNAs. Genome-wide association studies (GWAS) have identified many variants in these regulatory elements that can contribute to phenotypic diversity. However, the functional interpretation of these variants remains nontrivial due to the lack of chromatin contact information or limited contact resolution. Furthermore, the distribution and role of chromatin-associated RNAs in gene expression and chromatin conformation remain poorly understood. To address this, we first present a comprehensive interaction map of nuclear dynamics of 3D chromatin-chromatin interactions (H3K27ac BL-HiChIP) and RNA-chromatin interactions (GRID-seq) to reveal genomic variants that contribute to complex skeletal muscle traits.

**Results:**

In a genome-wide scan, we provide systematic fine mapping and gene prioritization from GWAS leading signals that underlie phenotypic variability of growth rate, meat quality, and carcass performance. A set of candidate functional variants and 54 target genes previously not detected were identified, with 71% of these candidate functional variants choosing to skip over their nearest gene to regulate the target gene in a long-range manner. The effects of three functional variants regulating *KLF6* (related to days to 100 kg), *MXRA8* (related to lean meat percentage), and *TAF11* (related to loin muscle depth) were observed in two pig populations. Moreover, we find that this multi-omics interaction map consists of functional communities that are enriched in specific biological functions, and GWAS target genes can serve as core genes for exploring peripheral trait-relevant genes.

**Conclusions:**

Our results provide a valuable resource of candidate functional variants for complex skeletal muscle-related traits and establish an integrated approach to complement existing 3D genomics by exploiting RNA-chromatin and chromatin-chromatin interactions for future association studies.

**Supplementary Information:**

The online version contains supplementary material available at 10.1186/s12915-022-01322-2.

## Background

Pig (*Sus scrofa*), a major farm animal worldwide, is one of the attractive models for genetic and genomic research due to its phenotypic diversity. Since its domestication about 10,000 years ago [1, 2], pigs have been subject to local adaptation and artificial selection, resulting in an obvious phenotypic split between different breeds in Europe and China [3]. For example, commonly used commercial lean breeds, such as Large White (LW) and Duroc (DU), have been strongly selected for skeletal muscle-related traits, including lean meat mass, daily gain, and feed conversion ratio. In contrast, local Chinese pig breeds such as Meishan (MS), perform relatively poorly on these traits, but have higher intramuscular fat content. In addition, pigs are biomedical models that are closer to humans than laboratory mice in terms of genetics, anatomy, and physiology [4]. Using pigs as a research model allows research on human diseases such as Duchenne muscular dystrophy [5], human type II diabetes [6], and heart xenotransplantation [7]. Therefore, great efforts including GWAS and genome-wide surveys have been made to identify genomic variants that contribute to complex phenotypic features in pigs over the past decade. However, the vast majority of these variants are present in noncoding regions, complicating the molecular interpretation of their mode of action.

Recent developments of the epigenomics make it possible to address these challenges. For example, open chromatin and histone modification play important roles in transcriptional regulation in both human and agricultural animals [8, 9]. Furthermore, extensive long-range chromatin interaction maps show that *cis*-regulatory elements contact each other in a hierarchical pattern in the three-dimensional genome [10]. Specifically, higher-order chromatin folds into megabase-sized topologically associating domains (TADs) that compartmentalize and insulate the genome [11–13]. Within TADs, regulatory gene expression programs were established in chromatin loops by bringing promoters and their distal regulatory elements into close physical proximity [14, 15]. Together, these advances contribute to an efficient approach to connecting GWAS leading signals to their target genes in complex traits. Our previous work has systematically characterized *cis*-regulatory elements in 12 tissues from four pig breeds and chromatin conformation in LW [16], which provides detailed comparative epigenetic data relevant to humans. However, global high-resolution mapping of GWAS leading signals to their target genes is still lacking, especially in the context of complex muscle-related traits.

Here we performed a systematic and deep analysis to identify the functional variants and to elucidate the gene regulatory mechanisms underlying the genetic associations reported in recent GWAS collections. We identified candidate functional variants from promoter-anchored active *cis*-regulatory elements interactome by integrating H3K27ac BL-HiChIP and GRID-seq. We identified 223 candidate functional single-nucleotide polymorphisms (SNPs) regulating 54 target genes related to 15 skeletal muscle traits and revealed the regulatory mechanisms of these SNPs. Furthermore, we observed the regulatory effects of 3 candidate functional SNPs and established functional networks based on chromatin and RNA interactions. Taken together, these results emphasize the value of creating high-resolution *cis*-regulatory interaction maps for understanding the mechanisms through which candidate functional variants affect the expression of genes related to skeletal muscle traits.

## Results

### Characterization of epigenetic landscape of skeletal muscle

**Fig. 1:**
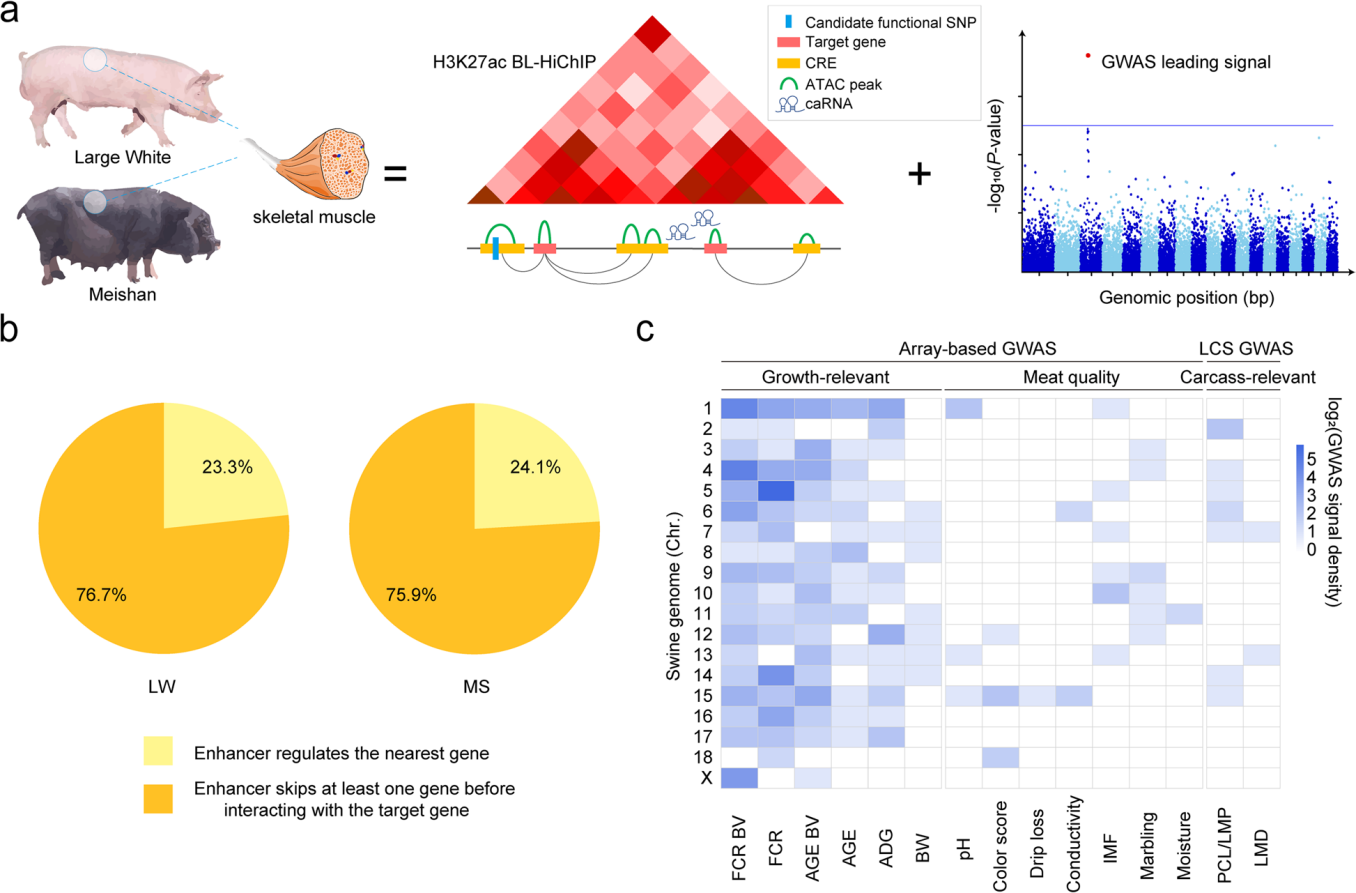
Study design and features of epigenetic landscape of skeletal muscle in LW and MS. **a** Integrated analyses based on enhancer-promoter interaction maps and GWAS leading signals. **b** Pie charts showing that 75–76% enhancers chose to skip over their nearest gene to regulate target genes in a long-range manner. **c** GWAS leading signals of 15 muscle-associated traits. The first category was growth-related traits, including feed conversion ratio (FCR), average daily gain (ADG), days to 100 kg (AGE), body weight (BW), and breeding values of FCR and AGE. The second category was meat quality-related traits, consisting of meat color score, conductivity, drip loss, marbling score, moisture, pH, and intramuscular fat (IMF). The last category was carcass-associated traits, including loin muscle depth at 100 kg (LMD) and lean meat percentage at 100 kg (LMP/PCL)

Regulatory elements often interact with genes over long genomic distances, thus preventing the correct identification of target genes and limiting the interpretation of functional noncoding variants of GWAS. In order to investigate the global epigenetic landscape of transcription networks and identify new associations of variants with target genes, we performed a multi-omics analysis of LW and MS skeletal muscle tissues (Fig. 1a). We used H3K27ac BL-HiChIP to identify chromatin interactions on active *cis*-regulatory elements. After read mapping and noise removal, approximately 200 million unique contact pairs for each breed were retained and assigned to a 5-kb fragment (Additional file 2: Table S1). High concordance was observed between replicates (Additional file 1: Fig. S1a and Additional file 2: Table S2). Approximately 85% of the loop anchors were found to be covered by H3K27ac ChIP-seq peaks (Additional file 1: Fig. S1b), and they were enriched with open chromatin (Additional file 1: Fig. S1c), indicating the reliability of loop anchors. A total of 103,511 and 84,723 chromatin loops were identified in LW and MS by FitHiChIP [17], respectively. We found that the LW chromatin interaction level was stronger on LW loop anchors than on MS loop anchors and that the MS chromatin interaction level was stronger on MS loop anchors than on LW loop anchors, which revealed breed-specific chromatin interactions in LW and MS (Additional file 1: Fig. S1d). We calculated the total contact counts of each detected loop, and about 40% of loops were supported by more than 20 contact counts (Additional file 1: Fig. S1e). In addition, the majority of interactions occurred within TADs (Additional file 1: Fig. S2a), suggesting the insulation role of TADs as a basic compartment for genome architecture [18]. The median interaction distance of both breeds was around 80 kb with most interaction distance ranging from 100 Kb to 1 Mb (Additional file 1: Fig. S2b and S2c). Moreover, about 75–76% of enhancers chose to skip over the nearest gene to regulate their target genes in a long-range interaction manner (Fig. 1b and Additional file 1: Fig. S2d). For example, the analysis of the best-known meat quality-associated gene *PRKAG3* indicated that the exact identification of target genes of any particular functional elements/variants might depend on detecting high-resolution chromatin interaction rather than seeking them from adjacent genes (Additional file 1: Fig. S2e).

We obtained 248 GWAS leading signals and 14 QTLs from the previously published datasets and classified them into three categories (Additional file 2: Table S3, S4, and S5). The first category was growth-related traits, including feed conversion ratio (FCR), average daily gain (ADG), days to 100 kg (AGE), body weight (BW), and breeding values of FCR and AGE. The second category was meat quality-related traits, consisting of meat color score, conductivity, drip loss, marbling score, moisture, pH, and intramuscular fat (IMF). The last category was carcass-associated traits, including loin muscle depth at 100 kg (LMD) and lean meat percentage at 100 kg (LMP/PCL). Specifically, we examined the distribution of aforementioned GWAS signals (Fig. 1c) and noticed that leading signals of meat quality and carcass-relevant traits were enriched in only several chromosomes, while leading signals of growth-related traits were extensively spread across the genome, indicating that growth traits were much more complex and might be modulated by a large number of weak- or moderate-effect variants [19].

Meanwhile, we used GRID-seq to identify chromatin-associated RNAs (caRNAs) and RNA-chromatin interactions. Most RNA–chromatin interactions were highly reproducible (Additional file 1: Fig. S3a) from the entire genome (Additional file 1: Fig. S3b) and from an enlarged view of a single chromosome (Additional file 1: Fig. S3c). In total, 1942 and 1488 caRNAs were identified from LW and MS by using uniquely mapped RNA-DNA read pairs, respectively. Comparative analysis revealed that 730 caRNAs were LW-specific; 276 caRNAs were MS-specific; and 1212 caRNAs were shared by both breeds (Additional file 1: Fig. S4a). Most RNA reads were mainly from various genic regions, indicating their origins of spliced transcripts, while an increased proportion of DNA reads resided in intergenic regions (Additional file 1: Fig. S4b), revealing the potential regulatory function of caRNAs through various binding loci. In addition, the first eigenvalue of Hi-C contact matrix exhibited a genome-wide correlation with GRID-seq DNA reads density (*R* = 0.84, *P* value < 2×10^−16^) (Additional file 1: Fig. S4c), suggesting an association of 3D genome spatial conformation and caRNA distribution. Each caRNA was classified into three types according to interaction range: local (±10 kb flanking their genes), *cis* (beyond local regions, but in the same chromosome), and *trans* (across different chromosomes). In both LW and MS, the majority of RNAs (both protein-coding and non-coding) exhibited a clear trend of proximal interaction near their transcription sites, indicating local interaction and *cis* interaction (Additional file 1: Fig. S3b and S4e). Interestingly, the caRNAs of protein-coding *TTN* were widely spread across the genome, implying a *trans* interaction pattern, which indicated their extensive participation in skeletal muscle functions (Additional file 1: Fig. S4d).

### Genome-wide chromatin interaction pattern in porcine skeletal muscle

**Fig. 2:**
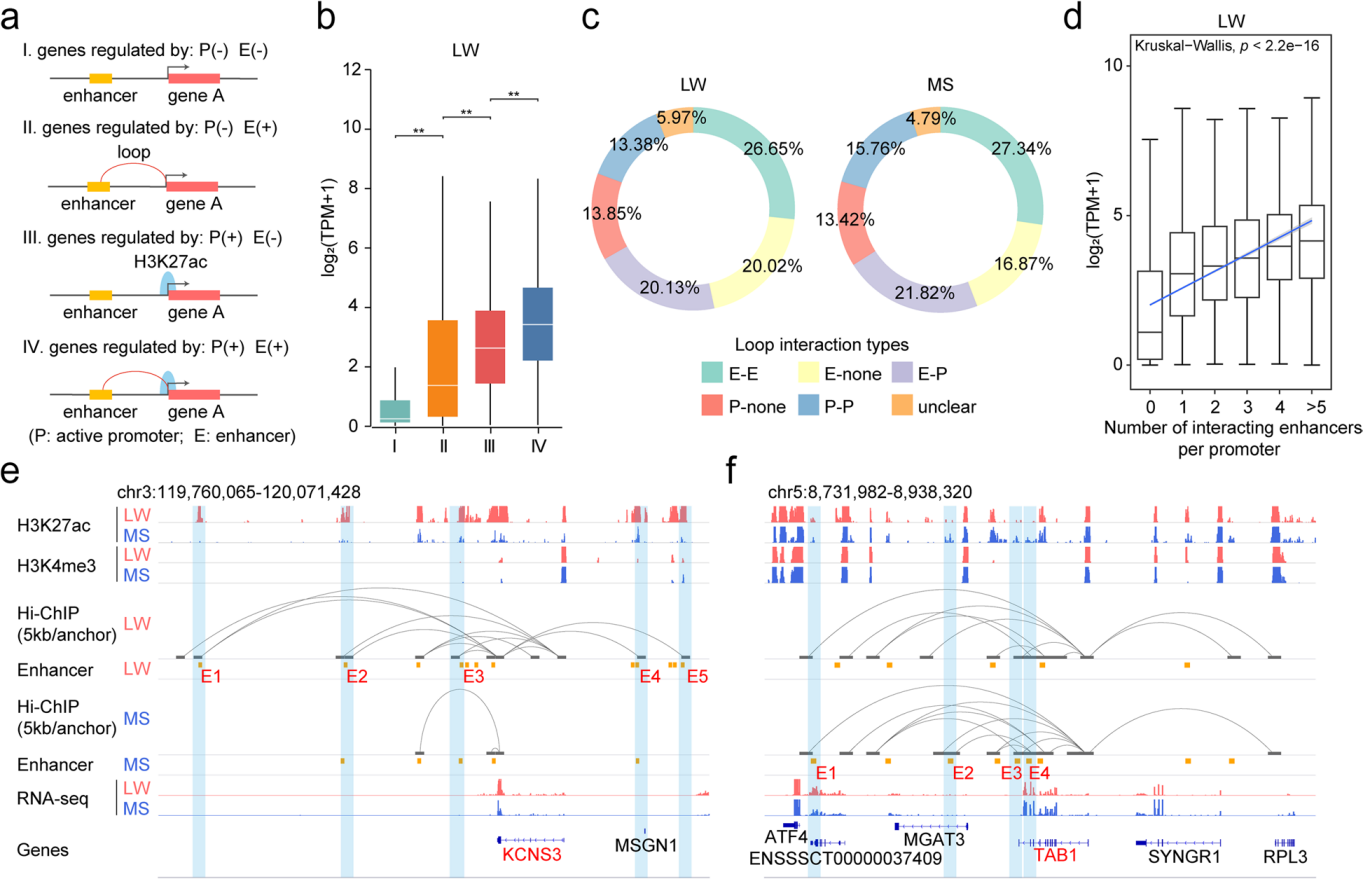
Comprehensive analyses of chromatin interactions and gene expression. **a** Genes with or without regulation by active promoters (P) and enhancers (E). Type I, genes without P and E; Type II, genes with E and without P; Type III, genes with P and without E; Type IV, genes with both E and P, respectively. **b** Expression of four types of genes in LW, type I (*n*=6381), type II (*n*=1308), type III (*n*=2163), and type IV (*n*=8314) by Mann-Whitney *U* single-tailed test with *P* values from left to right: 4.2×10^−131^, 1.0×10^−38^, and 6.9×10^−60^. **c** Donut plots (pie charts) of the percentages of loop interaction types. E–E, loops with both anchors covered with enhancers; P–P, loops with both anchors covered with active promoters; P–E, loops with one anchor covered with active promoter and the other anchor covered with enhancer; E-none, loops with just one anchor covered with enhancer; P-none, loops with just one anchor covered with active promoter; Unclear, loops with no anchor covered with active promoter and enhancer. **d** Expression of genes categorized according to the number of enhancers in LW. Linear regression of the mean gene expression level was performed (*P* value < 2.2 × 10^−16^, Kruskal−Wallis test). **e** Interaction of *KCNS3* with different counts of enhancers in LW and MS. **f** Interaction of *TAB1* with different counts of enhancers in LW and MS

We investigated genome-wide chromatin interaction pattern to reveal the effect of H3K27ac-mediated chromatin interactions at the transcriptional level. We compared the expression pattern of genes regulated by active promoters (P) and enhancers (E) (with or without) (Fig. 2a) and found that, as expected, the genes with P interactions exhibited higher expression levels than those without corresponding interactions, and the genes with E interactions also had higher expression levels than those without corresponding interactions. By contrast, the expression levels of genes with P interactions were significantly higher than those with E interactions (Fig. 2b and Additional file 1: Fig. S5a), which might be explained by the possibility that P (proximal elements) might exert a stronger influence than E (distal elements).

Further, to examine global chromatin characteristics mediated by H3K27ac loops, the type of loop-linked *cis*-regulatory elements were used to categorize chromatin interaction types. Chromatin interaction loops were highly enriched in active *cis*-regulatory regions, approximately 95% loops were linked to at least one active *cis*-regulatory element on either loop anchor in both breeds (Fig. 2c). E–E and E–P interaction types made up the two biggest chromatin interactions in both breeds, the sum of which accounted for almost half of total chromatin interaction, indicating that enhancers played a key role in complex transcription regulation. In addition, we found that the genes regulated by both E–P and P–P interactions were more abundantly expressed than the genes regulated by P-P alone, followed by E-E alone and basal genes (with no *cis*-regulatory elements detected) (Additional file 1: Fig. S5b), supporting our previous result that proximal *cis*-regulatory elements (P) might have more stronger effects on the regulation of single genes than distal *cis*-regulatory elements (E) acting as one of additive regulatory elements.

We further investigated the additive effects of enhancers on the regulation of gene transcription. In total, 7853 and 6925 genes interacting with one or more enhancers were identified from LW and MS, respectively (Additional file 1: Fig. S5c). The average number of enhancers interacting with one promoter was 1.58 and 1.43 in LW and MS (Additional file 1: Fig. S5d), which was consistent with the previous findings that one promoter could be regulated by multiple regulatory elements in humans [20, 21]. Multiple enhancers presented additive effects on the gene transcription regulation, a modest correlation was observed between the number of enhancers and the mean gene expression in each category of genes classified in terms of the number of interacting enhancers (*P* value < 2.2 × 10^−16^, Kruskal−Wallis test for linear regression) (Fig. 2d and Additional file 1: Fig. S5e). A case in point was *KCNS3* related to ion channel activity and potassium channel regulator activity [22], and this gene was found to interact with extra five enhancers in LW, resulting in its higher expression level in LW than in MS (Fig. 2e). Another example was *TAB1* involved in various intracellular signaling pathways such as TGF beta, interleukin 1, and WNT-1 [23], and its expression level was higher in MS than in LW due to the increased number of interacting enhancers (Fig. 2f).

### Loop interactions contribute to identifying breed-specific transcription regulation

**Fig. 3:**
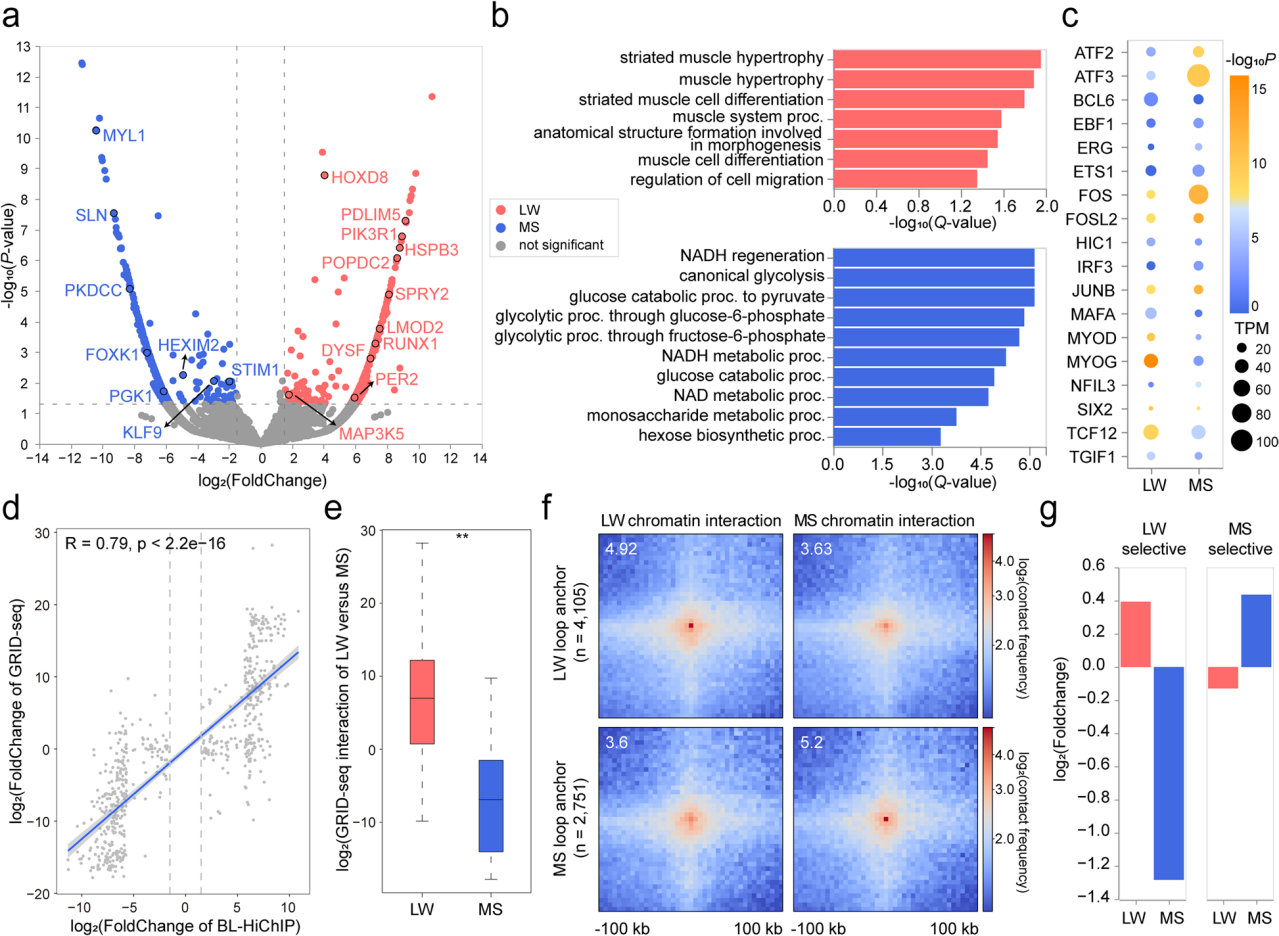
Differential chromatin-chromatin interactions and RNA–chromatin interactions. **a** Volcano plots showing chromatin loop up-regulated genes in LW and MS. **b** Top GO BP (biological process) terms enriched with LW and MS up-regulated genes by g:Profiler. Top enriched GO terms are ranked by the negative log_10_ (*Q*-value). **c** Enrichment of TF motifs on loop anchors of up-regulated genes in LW and MS. The color of each dot denotes the degree of enrichment (calculated as the cumulative binomial distribution by HOMER), and the size of each dot indicates the expression level of the corresponding TF. **d** Scatter plot showing that the differences in chromatin-chromatin interaction and RNA-chromatin interaction levels were positively correlated between LW and MS (Pearson correlation, *n* = 665 promoters). The trend line from linear regression is shown. **e** Change in RNA-chromatin interactions of differential loop-regulated promoters between LW (*n* = 328) and MS (*n* = 337), single-tailed Wilcoxon test with *P* values < 2.2×10^−16^. **f** Aggregate BL-HiChIP heatmap around differential caRNA-regulated genes and associated loop anchors based on RNA-chromatin interaction levels in LW (*n* = 4105) and MS (*n* = 2751). **g** Bar plot of LW up-regulated genes enriched in LW selective regions and not in MS selective regions, and vice versa for MS

We performed differential analysis through the comparison of LW versus MS, and identified 9598 genes regulated by loop interaction, of which 328 were up-regulated in LW and 337 were up-regulated in MS (|log_2_FC|>1.5, *P* value<0.05) (Fig. 3a). Gene ontology (GO) enrichment analysis (Fig. 3b) indicated that in LW, up-regulated genes such as *DYSF*, *LMOD2*, and *POPDC2* were enriched in muscle development GO terms mainly including striated muscle cell differentiation and muscle system process. Notably, *DYSF* is responsible for muscle membrane repair machinery during muscle degeneration [24], and its function loss is associated with adipogenic loss in muscular dystrophy [25]. In contrast, in MS, up-regulated genes were significantly enriched in GO terms associated with metabolism such as NADH regeneration and canonical glycolysis. For example, *SLN* is a key mediator of muscle thermogenesis and whole-body energy metabolism [26, 27].

In addition to regulation by chromatin loop interactions, gene expression profiles are simultaneously regulated by transcription factors (TFs). Therefore, we used HOMER [28] to identify TF motif enrichment on loop anchors of LW up-regulated and MS up-regulated genes (Fig. 3c). In LW, key TFs responsible for muscle structure development such as MYOG, MYOD and TCF12 were highly enriched in the loop anchors of upregulated genes. In contrast, in MS, key TFs responsible for muscle energy metabolism such as IRF3, a TF maintaining systemic glucose and energy homeostasis were enriched in the loop anchors of upregulated genes [29]. These results indicated potential synergistic regulatory roles of chromatin loops and TFs in gene transcription.

In addition, we assessed RNA-chromatin interactions on loop anchors of up-regulated genes in LW and MS. Differences in the levels of chromatin-chromatin interactions and RNA-chromatin interactions were positively correlated (Fig. 3d, e), suggesting that these two interactions might be coordinated in transcription regulation. Notably, this positive correlation was higher on differential loop-regulated promoters (*R*=0.79) (Fig. 3d) than on all loop-regulated promoters (*R*=0.45) (Additional file 1: Fig. S5f), suggesting that this potential coordination might be reinforced in actively functional genes. Furthermore, we performed differential RNA-chromatin analysis and identified a total of 11,397 caRNA-regulated genes, of which 838 were up-regulated in LW and 1238 were up-regulated in MS (|log_2_FC|>2, *P* value<0.05) (Additional file 1: Fig. S5g). We evaluated the chromatin-chromatin interaction on loop anchors in these caRNA-differentially-regulated genes. The results indicated that the chromatin interaction pattern on these representative loop anchors exhibited high consistency with the change of caRNA interaction (Fig. 3f). Our results were in line with the previous report that nascent caRNA-mediated RNA-chromatin interactions might be coordinated with chromatin-chromatin interactions for regulating gene transcription [30]. Next, to investigate the relation between genomic variation and breed-specific transcription regulation, we identified 320 and 481 selection regions (the intersection between the top 5% F_ST_ and top 5% XP-nSL measured regions) by comparing LW and MS, respectively, with European and Asian wild boars (Additional file 2: Table S6 and S7). We found that LW loop anchors of up-regulated genes were strongly enriched in LW selective sweep regions and not in MS selective sweep regions, and vice versa for MS (Fig. 3g). Taken together, chromatin-chromatin and RNA-chromatin interactions can reveal the breed-specificity in finely-tuned transcription network and provide epigenetic interpretation for genomic variation of complex traits in pigs.

### Enhancer-promoter interactions link candidate variants to target genes

**Fig. 4:**
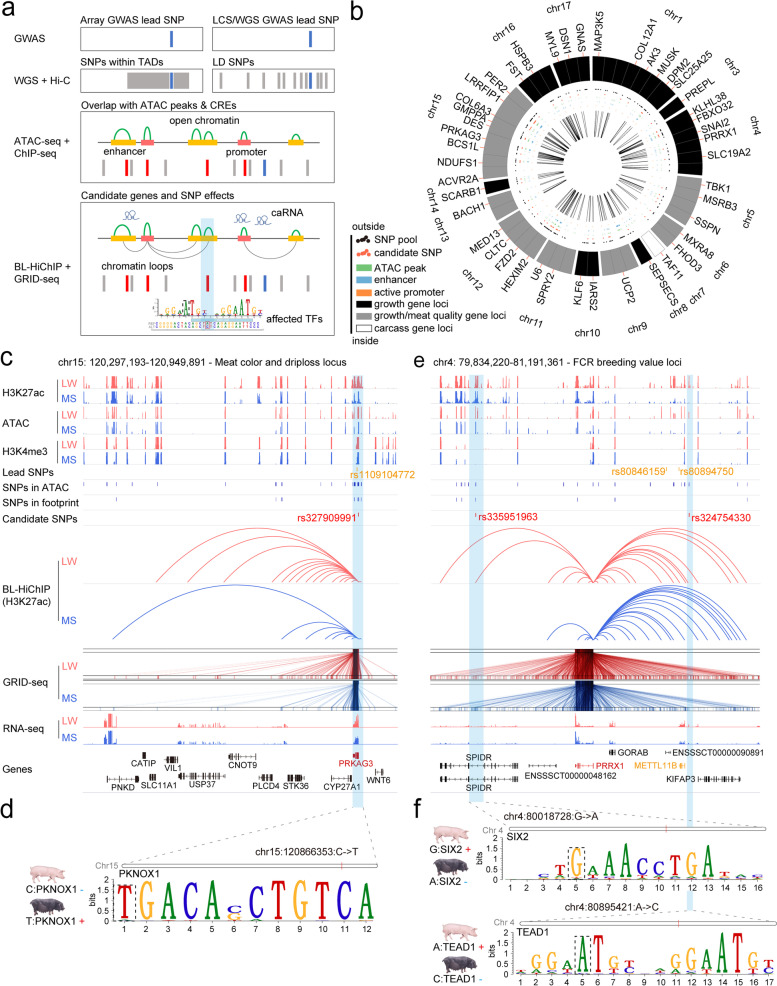
Multi-omics analyses of candidate functional SNPs and target genes. **a** The overall strategy for identification of candidate functional SNPs and their corresponding target genes: Firstly, SNPs located within loop-mediated CREs were screened. Secondly, the resultant SNPs located in ATAC peaks or footprints were further screened. Thirdly, the obtained SNPs with |ΔAF|≥0.5 (between commercial lean pig breeds and Chinese local pig breeds) were retained. Fourthly, the retained SNPs whose corresponding target genes were related to muscle traits and had intra-chromosomal RNA-chromatin interactions were screened. Fifthly, those SNPs which were predicted to affect TF binding were identified as final candidate SNPs. **b** Circos plot of 15 muscle-related traits, 54 target genes, and 223 candidate functional SNPs across the genome. Promoter track (orange), enhancer track (blue), ATAC peak track (green), and three categories of genes are labeled. **c** Example of *PRKAG3* in LW and MS. Red indicates newly-identified candidate functional SNPs and target genes in this study. Orange denotes previously predicted functional SNPs and target genes identified by GWAS. The blue bar highlights the candidate functional SNPs. **d** Predicted SNP affecting the motif of PKNOX1. **e** Example of *PRRX1* in LW and MS. Red indicates newly-identified functional SNPs and target genes in this study. Orange denotes previously predicted functional SNPs and target genes by GWAS. The blue bar highlights the candidate functional SNPs. **f** Predicted SNPs affecting the motifs of SIX2 and TEAD1

The high specificity of loop interaction allows to identify candidate target genes from GWAS leading signals for skeletal muscle-related traits. The GWAS leading signals are categorized into low- and high-marker density ones, and the former is mainly derived from array-based sequencing (IlluminaPorcineSNP50K Beadchip or IlluminaPorcineSNP60K Beadchip) with a density of approximately one SNP per 2×10^6^ bp, the latter is from low-coverage sequencing (LCS) with a density of approximately one SNP per 200 bp. Generally, the resolution and power of GWAS rely on the density of genetic markers. However, high-density marker panels are unavailable for most agricultural animals, which limits linkage disequilibrium (LD) analysis frequently conducted in human studies. Therefore, we employed two distinct strategies to obtain the associated SNPs of array-based GWAS and LCS-based GWAS leading signals to better accommodate the marker density of each approach (Fig. 4a). First, we used TAD to locate SNPs associated with array-based GWAS leading signals since TADs have been found to be fundamental transcriptional regulatory regions that can spatially insulate the genome, and *cis*-regulatory elements tend to interact with target genes within the same TAD. In contrast, we used the LD approach to obtain SNPs associated with high-density GWAS leading signals. After obtaining all the trait-related SNPs, we developed a 5-step strategy (detailed in the “Methods” section) to fine map potential functional SNPs (Fig. 4a). In addition, to investigate the power of the TAD approach, we compared the number of SNPs obtained by TAD and LD. Before the 5-step filtering, the TAD approach identified a candidate SNP pool containing 8292 SNPs and the LD approach identified a candidate SNP pool containing 632 SNPs, only 22.0% SNPs were exclusively identified by LD (Additional file 1: Fig. S6a). After the 5-step filtering, TAD approach identified 221 candidate functional SNPs and LD approach identified 23 candidate functional SNPs, only 2 SNPs were exclusively identified by LD (Additional file 1: Fig. S6a), which indicated that TAD approach could enlarge candidate SNP pool and minimize the probability of losing potential true signals.

In total, we identified 223 SNPs regulating 54 candidate genes related to 15 traits (Fig. 4b, Additional file 2: Table S8). Only 64 SNPs (28.7%) regulated the nearest gene, while 159 SNPs (71.3%) skipped over at least one gene to regulate their target genes (Additional file 1: Fig. S6b). Furthermore, 17% of SNPs were located on promoters, whereas 83% were on enhancers (Additional file 1: Fig. S6b). Altogether, this multi-omics approach identified novel trait-related genes and candidate functional SNPs. For example, the previously detected candidate target gene related to muscle physiology traits (such as glycogen content, meat color, and drip loss) was *PRKAG3* [31–33], which harbored one nonconservative substitution (rs1109104772, known as R200Q), in contrast, our models identified one novel candidate functional SNP (rs327909991) (Fig. 4c). SNP rs327909991 was located in the active promoter of *PRKAG3*, and this SNP was predicted to be able to enhance binding of TF PKNOX1 (Fig. 4d). Notably, PKNOX1 is able to exert major effects on the sensitivity of the glucose transport machinery to insulin and regulates energy metabolism in skeletal muscle [34, 35], which is consistent with our functional prediction. Another example is related to the breeding value of FCR (FCR BV), 30 SNPs were identified to be located on chromosome 4 in previous study, of which the two most significant SNPs were conjectured to be associated with *METTL11B* [36]. In this study, we identified a new target gene *PRRX1* (which acts as a transcriptional regulator of muscle creatine kinase), and two new associated candidate functional SNPs (rs335951963 and rs324754330). One new SNP was located within an enhancer 408.9 kb downstream from *PRRX1* and the other SNP was located in an enhancer 393.7 kb upstream from *PRRX1*, respectively (Fig. 4e). SNP rs335951963 was predicted to be able to disrupt the binding of SIX2*,* a TF involved in muscle energetic adjustment from oxidative to glycolytic capacities [37], and SNP rs324754330 was predicted to disrupt the binding of TEAD1*,* a TF which plays a critical role in regulating mitochondrial function in cardiomyocytes and its function loss could lead to a significant decrease in respiratory rates [38] (Fig. 4f). Altogether, the integration of GWAS data with multi-omics datasets can expand the understanding of both new target genes and relevant candidate functional SNPs.

### Effects of candidate functional SNPs in pig populations

**Fig. 5:**
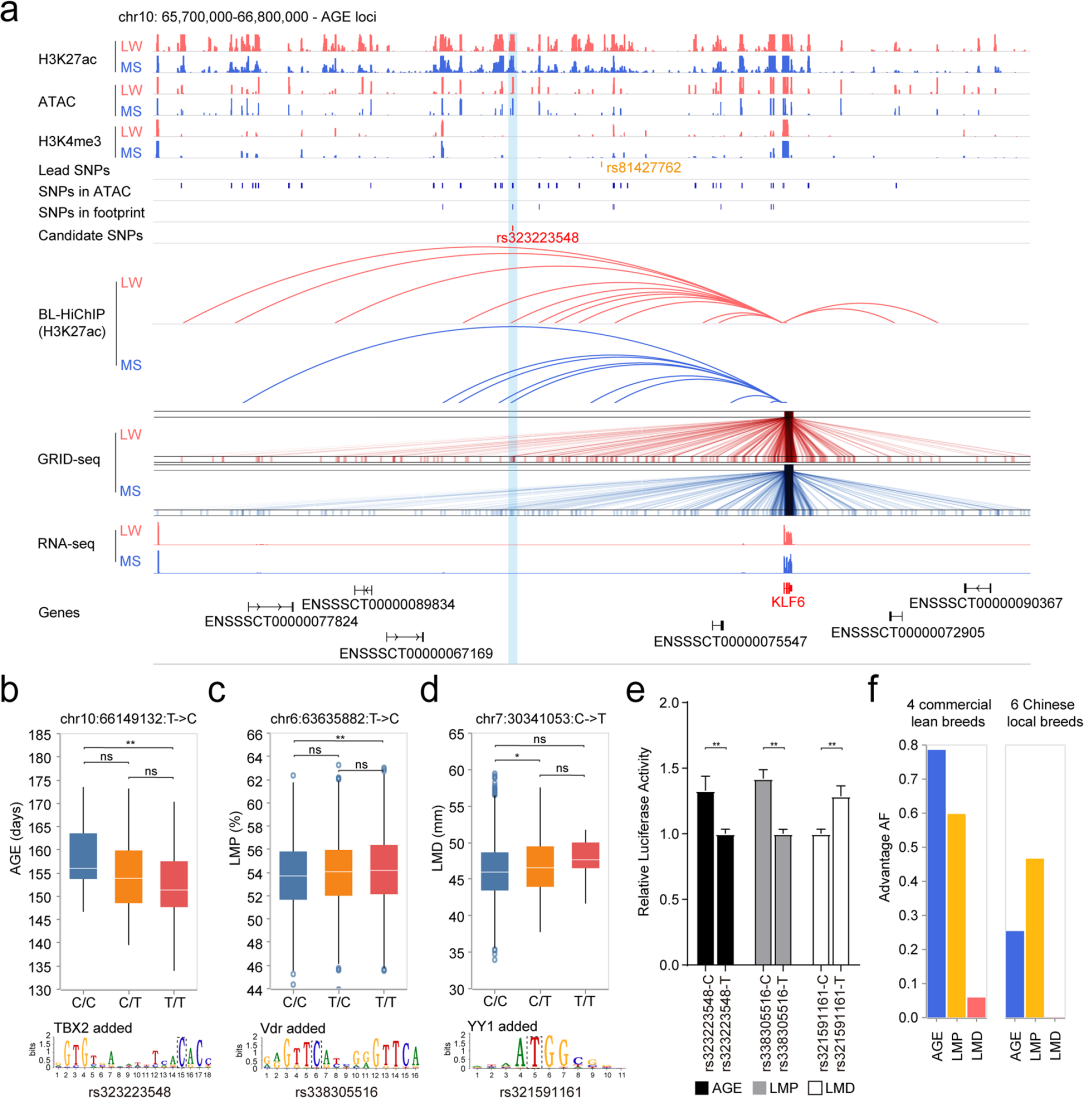
Validation of newly-identified candidate functional SNPs. **a** IGV plot of AGE trait regulated by candidate functional SNP based on multi-omics analysis. **b** Phenotypic differences of AGE (days to 100 kg) in a population of 182 LW individuals with different genotypes. The number of animals with different genotypes for AGE: *n*_C/C_ = 21, *n*_C/T_ = 31, and *n*_T/T_ = 130. Outliers are shown as individual dots. Statistical significance is determined by single-tailed Mann-Whitney *U* test. The predicted motif affected by the candidate functional SNP is shown at the bottom, and the locus of this SNP is highlighted by black dotted line. **c**, **d** Phenotypic differences of LMP (Lean meat percentage at 100 kg) and LMD (Loin muscle depth at 100 kg) in a population of 2869 Duroc individuals with different genotypes. The number of animals with different genotypes for LMP: *n*_C/C_ = 344, *n*_T/C_ = 1306, and *n*_T/T_ = 1135. The number of animals with different genotypes for LMD: *n*_C/C_ = 2456, *n*_C/T_ = 328, and *n*_T/T_ = 11. Outliers are shown as individual dots. Statistical significance is determined by single-tailed Mann-Whitney *U* test. The predicted motifs affected by the candidate functional SNPs are shown at the bottom, and the locus of SNPs is highlighted by black dotted line. **e** The alternative alleles exhibited significantly higher luciferase activity compared to the reference alleles. **f** Distribution of frequency of target alleles with enhanced phenotypic performance for AGE, LMP, and LMD traits

Based on phenotypic information of two pig populations (detailed in Methods), we observed the effects of three candidate functional SNPs. Specially, we investigated the effect of SNP rs323223548 on the AGE trait. This SNP was located in a super enhancer 339.2 kb upstream of its target gene *KLF6* on chromosome 10 (Fig. 5a), implying additional binding of TF TBX2 to this super enhancer (Fig. 5b). Pigs with T/T genotype (SNP rs323223548) exhibited a significant reduction in days to 100 kg compared to those with C/C genotype in Population I (*P* value = 4.2×10^−3^, single-tailed Mann-Whitney *U* test) (Fig. 5b). For carcass-related traits LMP and LMD, we observed the effects of two candidate functional SNPs in Population II. One SNP rs338305516 was located in the promoter of *MXRA8*, and pigs with T/T genotype exhibited significantly higher lean meat percentage (LMP) than those with C/C genotype (*P* value = 8.0×10^−3^, single-tailed Mann-Whitney *U* test), resulting in a potential Vdr binding to the above promoter (Fig. 5c and Additional file 1: Fig. S7a). The other SNP rs321591161 was located in the promoter of *TAF11*, and pigs with C/T genotype displayed significantly higher loin muscle depth (LMD) than those with C/C genotype, based on which we speculated that additional binding of TF YY1 might lead to stronger chromatin loop interaction on *TAF11* transcription regulation, thereby improving the LMD trait of pigs with T allele (*P* value = 2.0×10^−2^, single-tailed Mann-Whitney *U* test) (Fig. 5d and Additional file 1: Fig. S7b). Moreover, we performed a luciferase assay to further investigate the effects of these three SNPs. The results showed that the alternative alleles predicted above exhibited significantly higher luciferase activity than the reference alleles (Fig. 5e), suggesting that an altered genome could affect enhancer or promoter activity in porcine skeletal muscle.

We further calculated the allele frequency of the three aforementioned SNPs in four commercial lean pig breeds and six Chinese local pig breeds to assess their breeding potential. The target allele of each SNP with enhanced performance for each trait was plotted (Fig. 5f and Additional file 1: Fig. S8a). The bar chart showed that three target alleles exhibited higher frequency in commercial lean pig breeds than in Chinese local pig breeds. For AGE, target allele (T allele) of rs323223548 was almost fixed in Duroc, while less frequency was observed in LW, indicating a great improvement potential for LW. In contrast, Jinhua and Meishan showed the lowest frequency of this target allele. As for LMP trait, target allele (T allele) of Pietrain breed exhibited the highest (~90%) allele frequency among the commercial lean pig breeds, and the six Chinese local breeds displayed an average allele frequency around 40%. In terms of LMD trait, the non-target alleles (C allele) were fixed in all six Chinese local breeds, suggesting that introduction of new alleles from other commercial lean pig breeds might be required for breeding improvement of the LMD trait. All the four major commercial lean pig breeds except Pietrain exhibited approximately 10% or less of allele frequency on this SNP locus, indicating that long-term selection might be necessary for continuous improvement for loin muscle depth.

### Functional networks of chromatin-chromatin and RNA-chromatin interactions

**Fig. 6:**
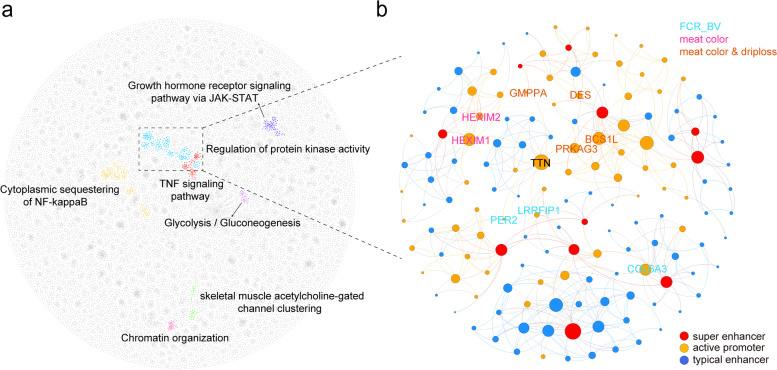
Functional communities of chromatin-chromatin and RNA-chromatin interaction network. **a** The network of all functional communities, some communities are highlighted and labeled. **b** One community harboring 9 genes out of 54 GWAS-detected genes (3 growth-associated genes and 6 meat quality-related genes)

We identified specific phenotype-genotype relations of 54 detected target genes for 15 skeletal muscle-associated traits mainly based on chromatin-chromatin interactions. Next, we investigated major caRNAs bound to the loop anchors of these 54 target genes and found that the composition and proportion of caRNAs differed between LW and MS (Additional file 1: Fig. S8b). Our findings provide a candidate RNA library for identification of key caRNAs involved in complex muscle-related traits. Interestingly, compared to other caRNAs, *TTN* was the most abundant among these 54 genes (Additional file 1: Fig. S8c), implying an extensive functional *trans* interaction in porcine skeletal muscle. We then established a 3D architectural network based on chromatin-chromatin and *TTN* mediated RNA-chromatin interactions. In total, 1,835 topologically-associated communities were formed within this network (Fig. 6a). Top 20 communities were analyzed by functional gene annotation. Among them, 9 communities showed enrichment in one or more GO terms or KEGG pathways (FDR<0.05) (Additional file 2: Table S9), suggesting that genes in the same community might be functionally related. Interestingly, one of the communities harboring 91 genes and 90 enhancers contained 9 out of the above 54 GWAS target genes. Of these 9 genes, 3 genes (*LRRFIP1*, *PER2*, and *COL6A3*) were associated with the trait of FCR breeding value, while the other 6 genes (*HEXIM1*, *HEXIM2*, *GMPPA*, *DES*, *BCS1L*, and *PRKAG3*) were associated with muscle physiological traits such as meat color and drip loss (Fig. 6b), further supporting the idea that genes within the same community tend to function in related biological processes. Taken together, our results provide a structural network for transcription regulation in which the GWAS-detected core genes and the interaction map-linked peripheral genes may jointly contribute to complex traits.

## Discussion

In pigs, the noncoding region composes the vast majority of the genome. GWAS and linkage analyses have identified many leading signals associated with complex traits, most of which are in the noncoding region. Therefore, the investigation of regulatory elements in the non-coding regions and the corresponding target genes is pivotal for deciphering the biological mechanisms of complex traits.

To explore the interaction between regulatory elements and their target genes, techniques for revealing 3D genomic features are needed. In this study, we employed high-resolution BL-HiChIP to construct maps of H3K27ac-centered chromatin interaction in LW and MS. Based on 5 kb resolution, our 3D genome map provides detailed topological information probably for the first time in pig skeletal muscles. Integrated analyses of enhancer-promoter interactions revealed that about 75% enhancers chose to skip over their nearest gene to regulate genes in a long-range manner, which challenge the commonly used ‘the nearest gene model’. Moreover, we found genes regulated by more elements in combined regulation modes (E–E, E–P, and P–P) exhibited higher expression levels than genes regulated by no or less regulatory elements. Meantime, the epigenetic difference also corresponded to the selective sweep signatures in LW and MS, revealing the influence of long-term artificial selection on finely-tuned transcription networks, thus contribute to complex phenotypic trait. However, it is difficult for BL-HiChIP to directly distinguish actively transcribing genes from inactive or transcriptionally poised genes, and BL-HiChiP can hardly identify inter-chromosomal chromatin interaction, thus limiting the ability of assessing *inter*-chromosomal chromatin interaction of actively transcribing genes contributing to complex traits. Therefore, GRID-seq was adopted to capture RNA-chromatin interaction to complementarily address these issues.

Eukaryotic genomes are pervasively transcribed to produce RNAs and also bound by a large number of RNAs, suggesting their mutual influence and interactions. In human and mice, some vital RNAs have been reported to play multiple roles. For example, *Neat1* induces H3K4me3 enrichment to activate gene transcription [39]. *Malat1* is localized in nuclear speckles and regulates gene expression by alternative splicing [40, 41]. *Jpx* regulates CTCF anchor site selection, thus influencing the formation of chromatin loops [42]. However, the global localization and functions of caRNAs in pig skeletal muscle have not been systematically characterized. In this study, we identified 1942 and 1488 caRNAs in LW and MS. We found that many RNA–chromatin interactions were positively correlated with chromatin–chromatin interactions, which is especially true with highly differentially regulated genes with breed-specificity. After identifying the target genes and candidate functional variants by GWAS and enhancer-promoter interaction maps, we further identified enriched caRNAs as well as their interactions with chromatin in response to differential transcription regulation between LW and MS. Notably, we identified a new transcript (Tx. 808) whose caRNAs were highly enriched in the above-mentioned 54 genes in MS, and this transcript is valuable for further studies of caRNA-mediated transcription regulation of commercial lean pig breeds and Chinese local breeds.

Most importantly, we observed extensive *trans* interaction between *TTN* and 54 GWAS target genes, indicating that transcriptional regulation of porcine skeletal muscle genes was associated with *TTN*-mediated *trans* interaction. In human, this *TTN*-mediated *trans* interaction was also observed during cardiogenesis, which led to genomic reorganization [43]. Based on the above observations, we established a transcriptional network in which functionally related genes such as meat quality- and growth-related genes can form communities through chromatin-chromatin interactions and RNA-chromatin interactions, thus co-regulating certain biological processes.

In conclusion, the network we built provides a model for identifying candidate genes related to certain muscle traits so as to provide a better explanation for the heritability contribution. Previous studies have reported that a quantitative phenotype model explaining complex traits is based on several core genes and a large number of peripheral genes, and co-regulation of core genes and peripheral genes might be crucial for explaining the genetic contribution of complex traits [19, 44]. Our results provide an approach for identifying a large number of peripheral genes by functional communities connected by chromatin and RNA interactions.

There are some limitations in combined analyses in this work. The first one is that the power of GWAS for identifying functional SNPs is limited due to the low SNP marker density [45]. The size and quality of the candidate SNP pool used for downstream multi-omics filtering rely on the density of SNP markers [46]. High SNP marker density can result in a relatively small SNP pool and less noise, thus more effectively identifying candidate functional SNPs. Furthermore, the GWAS leading signals were collected from different pig breeds and hybrids, but in our study, the BL-HiChIP and GRID-seq were only employed for LW and MS. Therefore, a breed-specific design of BL-HiChIP and GRID-seq experiment targeting different breeds might be needed to enhance the power of multi-omics analyses in further study.

## Conclusions

This study provides systematic fine mapping and gene prioritization based on 262 GWAS leading signals related to 15 complex pig skeletal muscle traits. Our 3D structure of the genome and related caRNAs sheds insight into the function of both distal and proximal regulatory elements. Our high-resolution chromatin interaction maps are valuable for identifying candidate target genes and corresponding *cis*-regulatory elements. The method and data presented in this study provide rich resources for functional genomics studies related to complex skeletal muscle traits.

## Methods

### Pig tissue collection

We used skeletal muscle tissues (longissimus dorsi) from two male piglets of two weeks old LW and MS. The samples were snap-frozen in liquid nitrogen. All experimental protocols were approved by the Ethics Committee of Huazhong Agricultural University (HZAUSW-2018-008).

### Publicly available data used in this study

All GWAS signals were collected from literature, as listed in Additional file 2: Table S3. Gene expression profiles, ChIP-seq (H3K4me3 and H3K27ac), ATAC-seq bigwig and peaks, *cis*-regulatory elements (CREs) and Hi-C results including TAD and TAD boundaries were derived from a previous study [16, 47]. The 78,334,029 SNPs from WGS analysis of four commercial lean pig breeds (LW, Pietrain, Landrace and Duroc, 185 individuals in total) and six Chinese local pig breeds (MS, Tongcheng, Rongchang, Jinhua, Erhualian and Bamei, 89 individuals in total) were obtained from ISwine database (http://iswine.iomics.pro/pig-iqgs/iqgs/index), further information and requests for this dataset of ISwine may be directed to the corresponding lead contacts, Yuhua Fu (yhfu2012@gmail.com) and Shuhong Zhao (shzhao@mail.hzau.edu.cn) [48]. All data were aligned to the Sus11.1 reference genome.

### Animal population, phenotypes, and genotyping

This study used two pig populations. Population I included a total of 182 LW pigs, and they were bred under the same feeding and management conditions in standard commercial pens. AGE was measured from 70 to 115 kg and then adjusted to 100 kg. Ear tissue samples were collected and preserved in 75% alcohol and stored in a refrigerator. Genomic DNA was extracted from frozen collected ear tissue samples using Tecan Freedom EVO NGS workstation with TIANGEN magnetic animal tissue genomic DNA kit. All the DNA samples had a concentration of ≥40 ng/μL and an amount of ≥1 μg. Genotyping of target SNPs was performed by sequencing. Population II included a total of 2869 Duroc pigs, and their genotyping was based on a Tn5-based low-coverage sequencing method. The phenotyping targeting LMD and LMP was performed by Yang et al. [49]. Both genotyping and phenotyping information of population II was deposited in GigaDB [50].

### Statistics and reproducibility

Statistical analyses were performed using Python 3.6.6. All of the statistical tests used are described in the relevant sections of the paper. *P* values were provided as exact values when possible; otherwise, they were reported as a range. The exact sample size (*N*) for each comparison group was provided in the figure and/or the legends. All the GRID-seq and BL-HiChIP libraries were generated and sequenced in duplicates. Mann-Whitney *U* single-tailed test results were presented in Fig. 2 b and Fig. 5 b–d and Additional file 1: Figure S5a-b. Kruskal-Wallis test results were shown in Fig. 2d and Additional file 1: Figure S5e. Wilcoxon single-tailed test results were displayed in Fig. 3e. Paired *T* test results were exhibited in Fig. 5e.

### BL-HiChIP library construction

Approximately 0.2 g of frozen muscle tissue was ground into a fine powder under liquid nitrogen conditions and fixed with cross-linking buffer (1% formaldehyde, 0.1 M NaCl, 1 mM EDTA, 0.5 mM EGTA, and 50 mM Hepes) for 15 min at room temperature. The cross-linking reaction was terminated by adding glycine at a concentration of 0.2 M. The sample was centrifugated at 2500 rpm for 10 min at 4°C, and then the sediment was collected and resuspended in tissue lysis buffer (containing 10 mM Tris-HCl pH 8.0, 10 Mm NaCl, 0.2% v/v Igepal CA630, 1× protease inhibitors), and incubated on ice for 30 min. The tissue was lysed and the nuclei were extracted. The impurities were removed with a 40 μL cell strainer to obtain high-purity nuclei. The resultant nuclei were resuspended in 1 mL SDS buffer (1×CutSmart and 0.5% SDS) and incubated for 10 min at 62°C, followed by the addition of 200 μL 10% TritionX-100 and incubation at room temperature for 10 min to neutralize the SDS. The nuclei were pelleted and washed with 1X Cutsmart buffer, and chromatin was digested with 1 U/μL AluI restriction enzyme at 37 °C for 7 h. The nuclei were collected, resuspended in 500 μL A -tailing solution, incubated for 1 h at 37 °C, pelleted again, resuspended again in 500 μL ligation solution added with biotinylated bridge linker, and incubated overnight at 16°C. Nuclei were fragmented by Covaris S220. Subsequently, 7.5 μg antibody (H3K27ac, Abcam Cat# ab4729) and 30 μL M-280 sheep anti-rabbit IgG Dynabeads (ThermoFisher, 11203D) were used to enrich the H3K27ac-mediated chromatin complexes. Chromatin complexes (ChiP-DNA) were purified and fragmented with Tn5. Streptavidin C-1 beads were used to pull down the DNA fragment with structure of DNA-linker-DNA. Library preparation and quality control were performed by the previously reported method [51].

### BL-HiChIP data processing

ChIA-PET2 software [52] was used to trim the linker for the BL-HiChIP sequencing data. The read pairs were aligned to the Sus11.1 reference genome using the BWA-MEM version 0.7.17 (r1188) [53] with a parameter of -SP5M. The invalid alignments were filtered with Pairtools (version 0.3.0) by removing duplicate reads, and only the reads identified as UU were retained. The aligned reads were assigned to one restriction fragment according to the reference genome and the restriction enzyme to separate the invalid ligation products from the valid pairs. Only valid pairs with two different restriction fragments were used for loop calling.

### Reproducibility analysis for BL-HiChIP

In order to detect the reproducibility of BL-HiChIP data, we first constructed a union set of significant loops in at least one replicate and then calculated the contact counts of these loops in two replicates with missing values replaced by zero. R function cor() was used to investigate the correlation between replicates. We merged samples of each breed for the subsequent analyses. In addition, we also used HiCRep [54] to assess the reproducibility of our BL-HiChIP data. By smoothing sparse matrix and adopting a stratum-adjusted correlation coefficient (SCC), we measured the reproducibility based on three different resolutions: 5 kb, 10 kb, and 40kb.

### Loop calling

FitHiChIP [17] was used to identify peak-to-all interactions using the peaks obtained from independent H3K27ac ChIP-seq. Genome-wide intrachromosomal pairs with an interaction distance of 5 kb-2 Mb were retained. The false discovery rate (FDR) values for each pair were calculated with default set. Bias correction was performed using coverage-specific bias at 5-kb resolution.

### Differential analysis of BL-HiChIP loop calls and visualization

We used an approach similar to calling SIP [55] to annotate loop-mediated genes in each breed. For each active promoter, we calculated the cumulative interaction score, defined as the sum of −log_10_FDR for interaction intensity. We then performed quantile–quantile normalization under the assumption that all the signals were identically distributed across all samples. Next, we used DEseq2 [56] to identify differentially regulated genes between LW and MS. We used Coolbox [57] for BL-HiChIP heatmap visualization and Coolpup.py [58] for pile-up visualization.

### GO enrichment analysis

For gene function analysis, we used Ensembl Biomart to transfer swine gene IDs into human IDs, and then uploaded to g:Profiler (https://biit.cs.ut.ee/gprofiler/gost) to obtain GO:BP functioning profiles.

### Transcription factor motif enrichment analysis

For differential transcription factor binding analysis, a 200-bp window was taken from open chromatin peaks on loop anchors of differential loop-mediated genes. Open chromatin peaks of these two breeds were used as background for each other.

### Footprint analysis

We used two software for footprint analysis. TOBIAS [59] was used for correction of insertion bias at Tn5 transposase sites with the command of ‘ATACorrect’. The ‘ScoreBigwig’ command was used to calculate a continuous footprinting score at all sites. To predict specific transcription factor binding, the footprinting scores were combined with vertebrate motif library JASPAR2018.

We also used Wellington [60] for footprint analysis. The broad ATAC peaks were determined using MACS2 [61]. Peaks with *P* value less than 10^−5^ were used to identify footprints based on the “wellington_footprints.py” script with a parameter of “-A” and a threshold of *P* value<10^−20^.

### Selective sweep analysis

In this study, we combined genetic differentiation coefficient F_ST_ and haplotype composition difference XP-nSL to detect genome-wide selection signals in LW and MS. Detailed methods are described in previous research [62].

### Target SNP identification based on GWAS signals

GWAS signals were listed in Additional file 2: S4 and S5. Since GWAS was performed by IlluminaPorcineSNP50K Beadchip or IlluminaPorcineSNP60K Beadchip, we first transferred Sus10.2 SNP loci information into Sus11.1 loci. To improve the quality and lessen the noise of GWAS leading signal-associated SNP pool, we used two strategies to identify candidate SNPs based on GWAS signals. Specifically, for low-coverage sequencing (LCS)-based GWAS, we used a linkage disequilibrium (LD) score ≥0.5 to identify the SNPs associated with GWAS leading signals, and for array-based GWAS, we used TAD to identify the SNPs associated with GWAS leading signals. The specific screening criteria and steps of target SNPs based on GWAS signals were as follows. Firstly, SNPs located within loop-mediated enhancers or active promoters were screened. Secondly, the resultant SNPs located in ATAC-seq peaks or footprints were further screened. Thirdly, the obtained SNPs with the absolute value of delta allele frequency (ΔAF=mean AF of four commercial lean pig breeds - mean AF of six Chinese local pig breeds) were retained (|ΔAF|≥0.5). Fourthly, the retained SNPs whose corresponding target genes were related to muscle traits and had intra-chromosomal RNA-chromatin interactions were screened. Fifthly, those SNPs which were predicted to affect TF binding were identified as final candidate SNPs.

### Effects of SNP on TF binding

We assessed the matching status of the screened SNP with known transcription factor binding sites by R package motifBreakR [63], and SNP whose effects were “strong” was kept and investigated.

### Luciferase activity assay

The reporter gene vector pGL3-Basic vector was used for promoter activity validation in this experiment. The validated promoter sequence was cloned to the upstream of the reporter gene, and the promoter activity was determined by testing the activity of the reporter gene. The reporter gene vector pGL3-promoter vector was used for enhancer activity validation in this experiment. The validated enhancer sequences were cloned to the upstream of the reporter genes, and the enhancer activity was determined by testing the activity of the reporter gene. Luciferase activity was detected after 48 h post-transfection in 3D4/21 cells.

### Construction of GRID-seq library

GRID-seq was performed by the method reported by Li et al. [64]. Approximately 0.2 g of frozen muscle tissue was ground into a fine powder under liquid nitrogen conditions and further fixed in 1% formaldehyde for 10 min. Samples were permeabilized in 1 mL equilibrium buffer (0.2% NP-40, 5 mM Tris-HCl pH 7.5, 10 mM NaCl, and 1× protease inhibitor cocktail) for 15 min on ice with brief centrifugation. Samples were incubated in 300 μL of Cutsmart buffer (0.5% SDS) for 10 min at 62 °C, added with 50 μL Triton X-100 (10%) to quench the SDS, and chilled on ice to prepare nuclei. The prepared nuclei were collected through brief centrifugation, washed twice with 1× Cutsmat Buffer, resuspended in 500 μL AluI solution (1× Cutsmart Buffer, 1% Triton X-100,1 U/μL RiboLock, 0.5 U/μL AluI (NEB), and 1× protease inhibitor), and incubated at 37 °C for 4 h to digest chromatin. After digestion, DNA ends were added with dA tailing module, and then repaired through DNA/RNA 5′ phosphorylation and 3′ dephosphorylation using PNK (NEB) at 37 °C for 1.5 h.

To ligate in situ linkers to RNA, RNA ends were ligated to an oligonucleotide “bridge” molecule containing a 5′-adenylated ssDNA overhang using 4U/μL T4 RNA Ligase 2-truncated KQ (NEB) and incubated at 25 °C for 3 h. To ligate in situ linkers to DNA, nuclei were collected, washed twice with 800 μL of 1× DNA Ligase Buffer (NEB) to remove free linkers, resuspended in 1 mL of DNA Ligation Solution (0.2 U/μL RiboLock, 1× DNA Ligase Buffer, 1 mg/mL BSA, 1% Triton X-100, and 1 U/μL T4 DNA Ligase (NEB)), and incubated overnight at 16 °C. Subsequently, the nuclei were washed, and the RNA strand was stabilized by first-strand synthesis of the RNA through the extension of the bridge by Bst 3.0 polymerase.

Crosslinking reversal of and DNA/RNA purification were performed. The pellets were resuspended in 300 μL proteinase K solution (15 mM EDTA, 10 mM Tris-HCl, pH 7.5, 0.5% SDS, and 1 mg/mL proteinase K (Ambion)), and incubated at 65 °C for at least 2 h in Thermomixer. After being added with 300 μL solution Phenol:Chloroform:Isoamyl Alcohol (pH 8.0, Thermo Fisher), total DNA/RNA were precipitated with solution (2 μL of GlycoBlue, 30μL of 3 M NaOAc (pH 5.5), and 300 μL of 100% isopropanol) on ice for at least 2 h, followed by centrifugation for 30 min at 16,000*g*. Total DNA/RNA was dissolved in 51 μL of H_2_O to obtain a total amount of ~10 μg DNA/RNA.

Biotin-labeled DNA/RNA was pulled down. The 50 μL DNA/RNA was added to M280 streptavidin magnetic beads, washed for three times with 1× B&W Buffer (5 mM Tris-Cl pH 7.5, 0.02% Tween-20, 0.5 mM EDTA, 1 M NaCl), and incubated for 45 min at room temperature, washed again with 500 μL of 1×B&W Buffer for 5 times, and resuspended in 100 μL freshly prepared 150 mM NaOH at room temperature for 10 min, followed by centrifugation. Afterwards, the supernatant was collected to a 1.5 mL tube and neutralized with 11 μL 10 × TE buffer and 6.5 μL of 1.25 M acetic acid. Single-stranded DNA (ssDNA) was precipitated in solution containing 2 μL of GlycoBlue, 10 μL of 3 M NaOAc (pH 5.5), and 100 μL of 100% isopropanol. Second strand synthesis reaction was performed by adding 0.5 μL of 10mM dNTP and 5 U klenow Exo-(NEB) at 37°C for 1 h. Next, the synthesized double-stranded DNA was digested with MmeI buffer (5 pmol SAM(NEB) and 4 U MmeI) at 37°C for 1 h. After MmeI digestion, DNA was extracted and purified, the 84bp target band was cut and used for Illumina TrueSeq library construction.

### GRID-seq raw data processing

After sequencing, reads from each library were trimmed, and those with a minimum length of 79 bp were retained. To precisely obtain reads and minimize the loss of reads with the full linker sequence absence due to sequencing and/or PCR errors, we adopted a multi-step strategy: (1) The reads that contained the core 12 bp seed sequence in linker were retained; (2) MmeI motifs were used to define linker boundaries, and (3) linker orientation was used to determine the RNA or genomic DNA origin of a read. With this strategy, paired DNA-RNA reads with length ranging from 17 bp to 23 bp were obtained. For each captured RNA read (cDNA), we used its reverse complement sequence as the formal read, and thus this read had the exact same sequence with the RNA product sequence. All paired reads were assigned to unique paired IDs. All clean read pairs were aligned separately to the pig reference genome (Sus11.1) using Bowtie2 [65] with the parameter “–local.” The read pairs that contained ambiguous mapping information were removed by SAMtools [66] using the parameter “-Sbq 2.”

### GRID-seq pipeline and downstream analyses

The chromatin-enriched RNAs were identified, the non-specific background was constructed, and specific RNA–chromatin interaction was characterized by the method reported by Li et al. [64].

R package “ggplot2” and “ggtern” were used to plot the ternary plot, in which each dot represented one chromatin-enriched RNA. The size of each point was proportional to the number of RNA reads at log_2_ scale. The position of each point in the triangular coordinates reflected the relative percentages of local, *cis*, and *trans* modes of RNA interaction.

Circos [67] plots were used to represent *trans* RNA interactions across the genome. The links were based on the background-corrected RNA-DNA interaction. The outer circle of histograms was plotted based on densities of the active promoters, typical enhancers, and super-enhancers in the 1Mb bin.

### Fruchterman-Reingold model visualizes complex networks of chromatin interactions

We used Fruchterman-Reingold model developed by Peter Eades to visualize the chromatin-chromatin and RNA-chromatin interactions across the genome [68]. This model aims to reduce the intersection of edges in the layout and keep the length of edges as consistent as possible. Through continuous iterations, the whole layout reached dynamic equilibrium and tended to be stable. The total energy of the system was calculated according to the following formula.

$${\boldsymbol{E}}_{\boldsymbol{s}}=\sum_{\boldsymbol{i}=\mathbf{1}}^{\boldsymbol{n}}\sum_{\boldsymbol{j}=\mathbf{1}}^{\boldsymbol{n}}\frac{\mathbf{1}}{\mathbf{2}}\boldsymbol{k}{\left(\boldsymbol{d}\left(\boldsymbol{i},\boldsymbol{j}\right)-\boldsymbol{s}\left(\boldsymbol{i},\boldsymbol{j}\right)\right)}^{\mathbf{2}}$$

where, *d*(*i*,*j*) represents the Euclidean distance between the two points; *s*(*i*,*j*) represents the natural length of the spring; and *k* is the elasticity coefficient.

## Supplementary Information

**Additional file 1: Figure S1.** Reproducibility, library quality and basic statistics of BL-HiChIP. **Figure S2.** Statistics of chromatin-chromatin interactions captured by BL-HiChIP. **Figure S3.** Correlation and reproducibility between GRID-seq replicates. **Figure S4.** RNA–chromatin interactions captured by GRID-seq. **Figure S5.** Chromatin loops affecting transcription regulation. **Figure S6.** Comparison between TAD and LD approaches and statistics of newly-identified 223 SNPs. **Figure S7.** IGV plot of two candidate functional SNPs associated with LMP and LMD traits. **Figure S8.** Allele frequency of three SNPs and major caRNAs associated with 54 GWAS target genes.

**Additional file 2: Table S1.** Sequencing information of BL-HiChIP data. **Table S2.** Reproducibility of BL-HiChIP replicates. **Table S3.** GWAS literatures used in this study. **Table S4.** GWAS leading signals used in this study. **Table S5.** QTL information used in this study. **Table S6.** Information of LW selected regions. **Table S7.** Information of MS selected regions. **Table S8.** Information of 223 candidate functional SNPs. **Table S9.** GO or KEGG of top 20 communities.

## Data Availability

All data generated or analyzed during this study are included in this published article, its supplementary information files, and publicly available repositories. The datasets are available in the NCBI Gene Expression Omnibus under the accession number GSE189397.
