# Supplementary material for: Enhancer-promoter interaction maps provide insights into skeletal muscle-related traits in pig genome
Source: BMC Biol. 2022 Jun 9;20:136. doi: 10.1186/s12915-022-01322-2 (PMC9185926; doi:10.1186/s12915-022-01322-2)
Supplement: Supplementary file 1 — Additional file 1: Figure S1. Reproducibility, library quality and basic statistics of BL-HiChIP. Figure S2. Statistics of chromatin-chromatin interactions captured by BL-HiChIP. Figure S3. Correlation and reproducibility between GRID-seq replicates. Figure S4. RNA–chromatin interactions captured by GRID-seq. Figure S5. Chromatin loops affecting transcription regulation. Figure S6. Comparison between TAD and LD approaches and statistics of newly-identified 223 SNPs. Figure S7. IGV plot of two candidate functional SNPs associated with LMP and LMD traits. Figure S8. Allele frequency of three SNPs and major caRNAs associated with 54 GWAS target genes. [file 12915_2022_1322_MOESM1_ESM.docx]

**Additional file 1**

**Figure S1.** Reproducibility, library quality and basic statistics of BL-HiChIP. **Figure S2.** Statistics of chromatin-chromatin interactions captured by BL-HiChIP. **Figure S3.** Correlation and reproducibility between GRID-seq replicates. **Figure S4.** RNA–chromatin interactions captured by GRID-seq. **Figure S5.** Chromatin loops affecting transcription regulation. **Figure S6.** Comparison between TAD and LD approaches and statistics of newly-identified 223 SNPs. **Figure S7.** IGV plot of two candidate functional SNPs associated with LMP and LMD traits. **Figure S8.** Allele frequency of three SNPs and major caRNAs associated with 54 GWAS target genes.

**
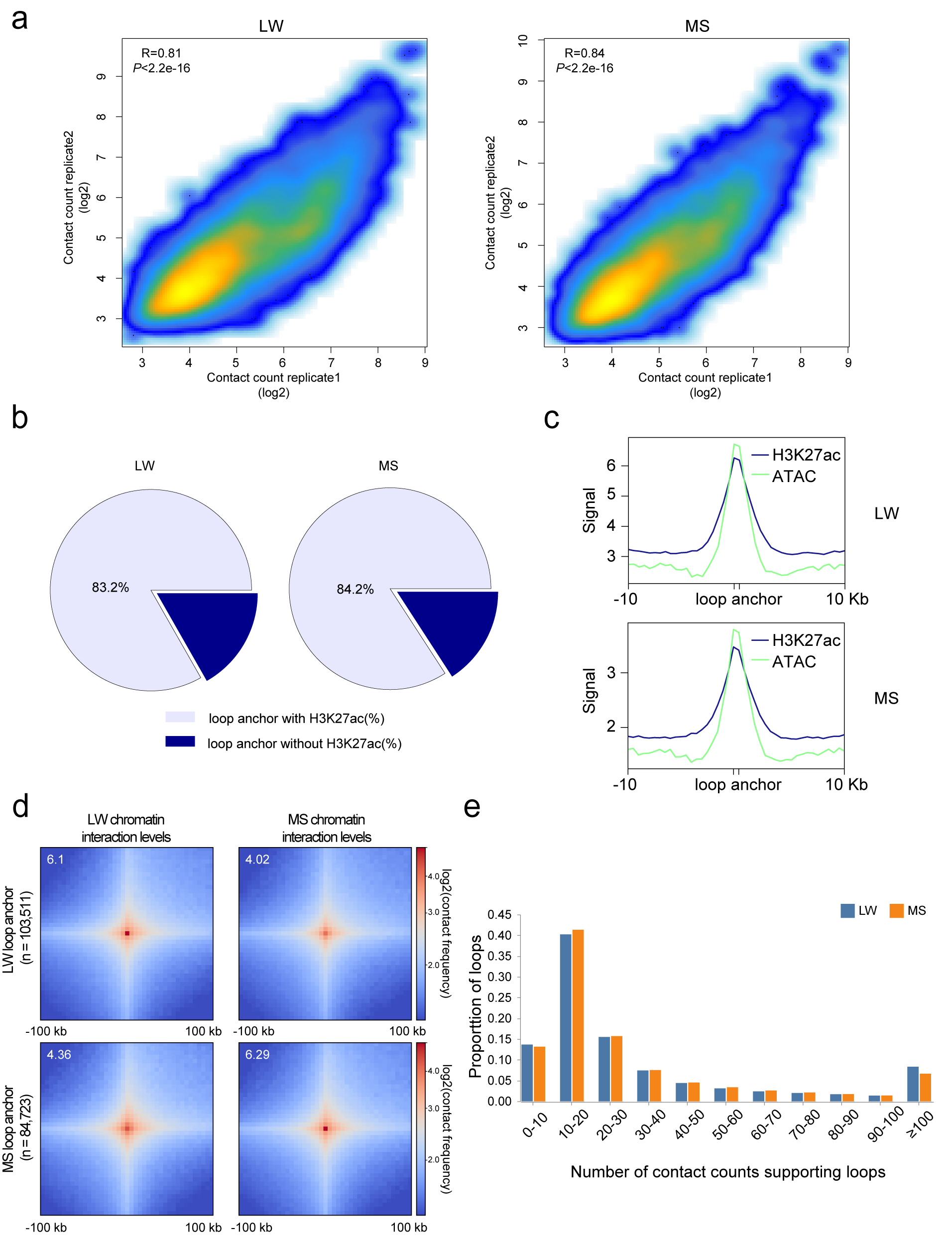
**

**Figure S1.** Reproducibility, library quality and basic statistics of BL-HiChIP. (**a**) Correlation between BL-HiChIP libraries of LW and MS. (**b**) Pie charts showing the ratio of loop anchors covered by H3K27ac ChIP-seq peaks. (**c**) Loop anchors enriched with H3K27ac and open chromatin signal. (**d**) Aggregate BL-HiChIP map of all detected loops showing breed-specificity of chromatin interactions between LW and MS. (**e**) Bar plot showing the distribution of contact count supporting loops.


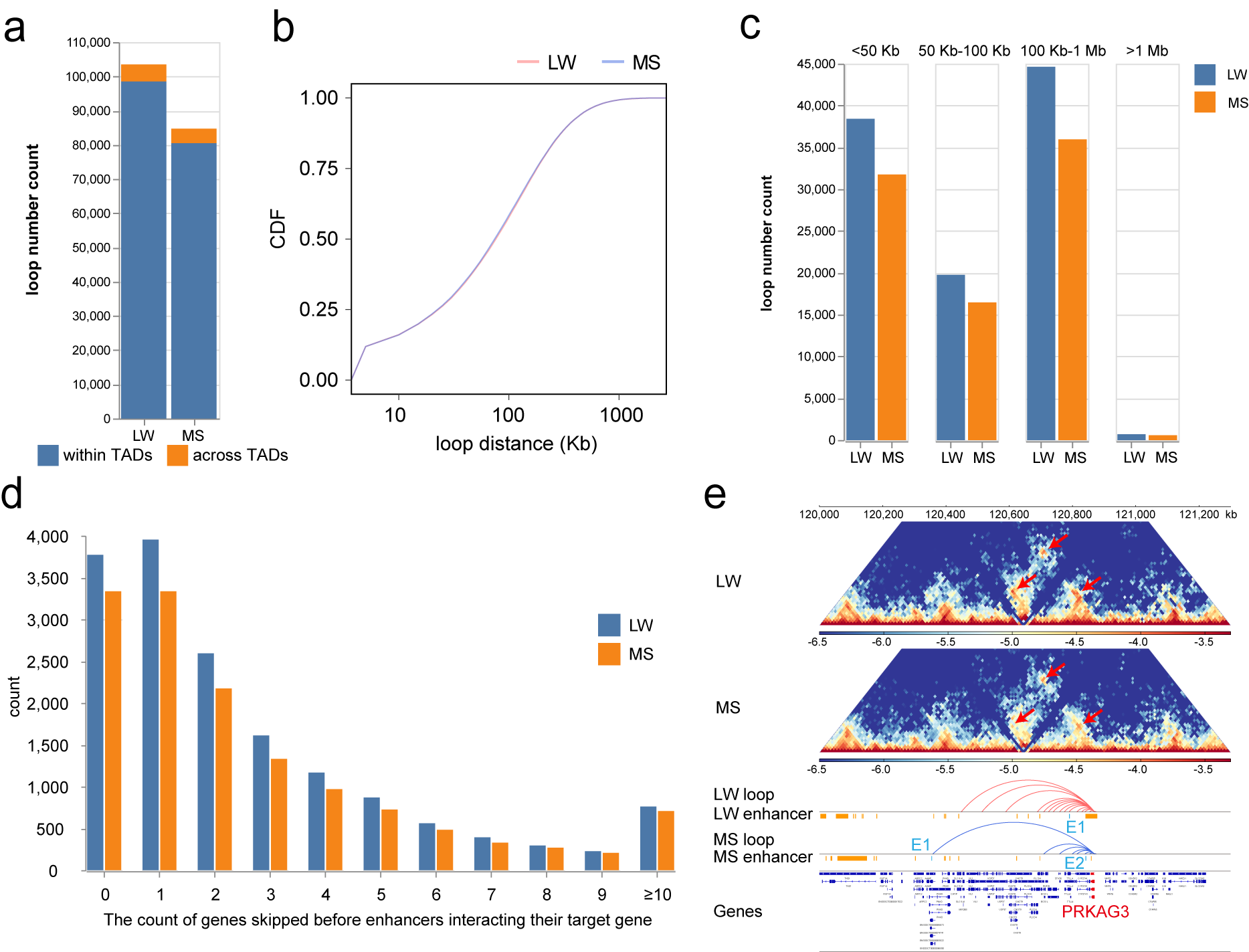


**Figure S2.** Statistics of chromatin-chromatin interactions captured by BL-HiChIP. (**a**) Proportions of loop interactions occurring within/across TADs in LW and MS. (**b**) Cumulative distribution function plot of loop interaction distances in LW and MS. (**c**) Summary of the number of loop interactions with variable distances in LW and MS. (**d**) Bar plot exhibiting the number of genes skipped before one enhancer could interact with their target gene. (**e**) *PRKAG3* as an example showing that enhancers chose to skip over their nearest gene to regulate the target gene in a long-range manner. In LW, E1 (typical enhancer, chr15:120,787,229-120,789,229) skipped two genes (*TTLL4* and *CYP27A1*) to regulate *PRKAG3*. In MS, E1 (typical enhancer, chr15:120,353,075-120,355,075) skipped 15 genes to regulate *PRKAG3*, and E2 (typical enhancer, chr15:120,838,862-120,840,862) skipped one gene (*CYP27A1*) to regulate *PRKAG3*.

**
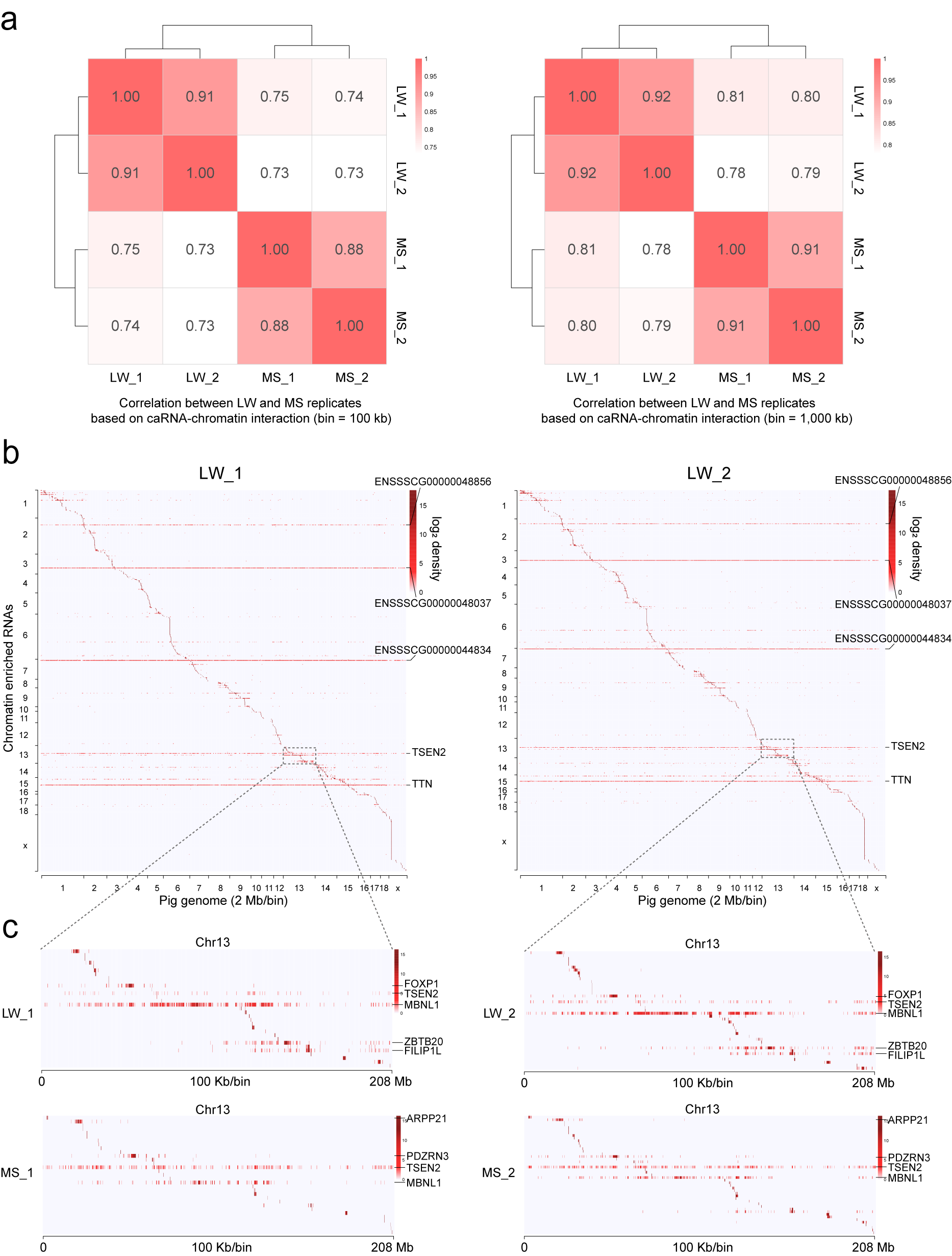
**

**Figure S3.** Correlation and reproducibility between GRID-seq replicates. (**a**) Correlation between GRID-seq replicates based on RNA-chromatin interaction across the genome. (**b**) Heatmap displaying caRNAs across the whole pig genome of LW rep1 and LW rep2. Row: caRNAs. Column: pig genome in 2-Mb resolution. Major *trans* caRNAs are labeled on the right. (**c**) Enlarged representative regions boxed in figure b, showing detailed *cis* RNA–chromatin interaction profiles on chr13 (100 Kb/bin). Some caRNAs are labeled on the right.


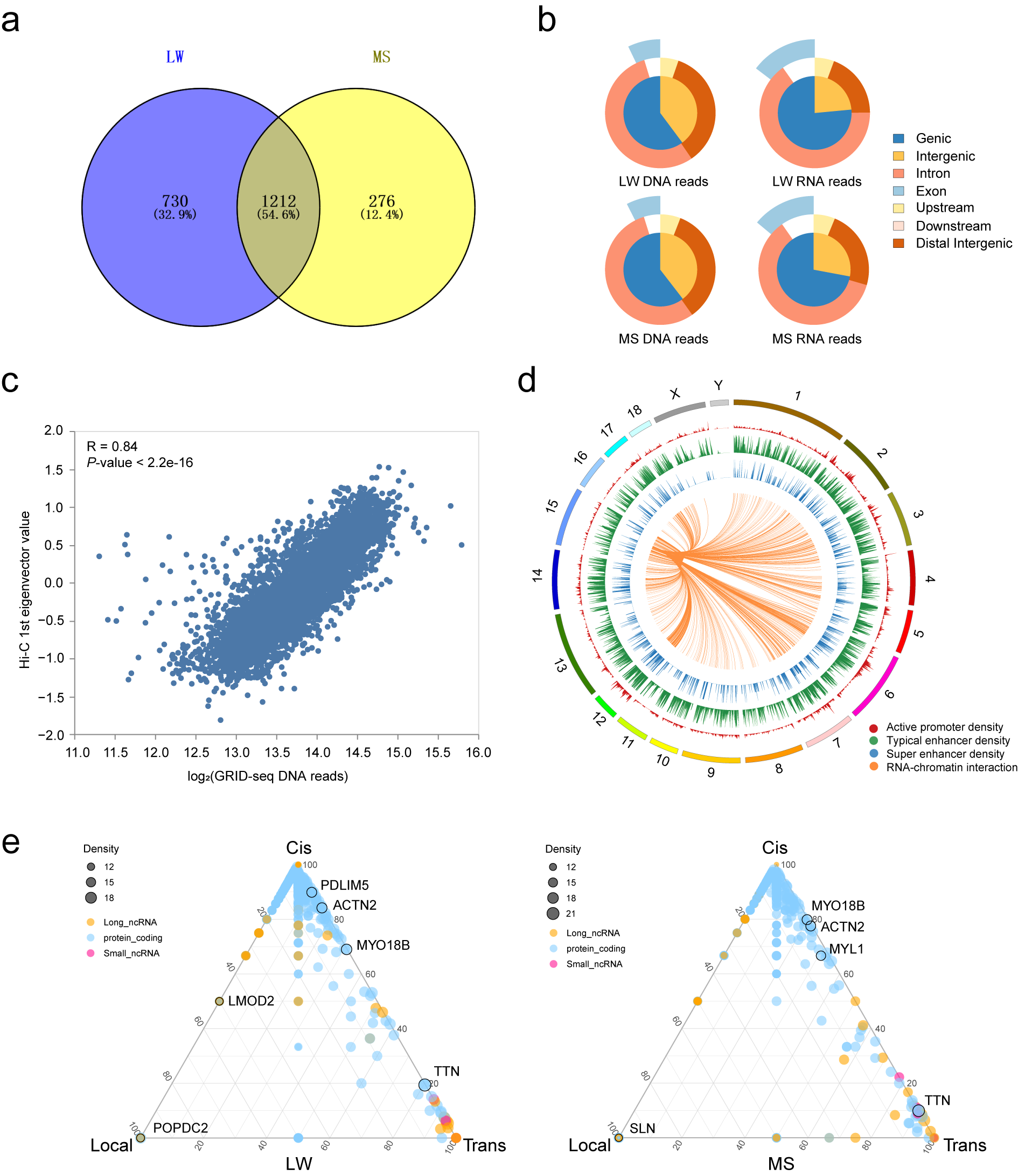


**Figure S4.** RNA–chromatin interactions captured by GRID-seq. (**a**) Overlap of caRNAs between LW and MS, illustrating breed-specificity of caRNAs. (**b**) Venn-pie plot illustrating the genomic distribution of uniquely-mapped RNA/DNA reads in LW and MS. (**c**) Scatterplot of Hi-C first eigenvector (y axis) and log_2_(GRID-seq DNA reads) (x axis) on every 500 kb genomic bin (dot) of the pig genome. (**d**) Circos plot depicting *TTN* RNA-chromatin interaction across the genome in LW. (**e**) Ternary plots of caRNAs features, showing the proportion of individual chromatin-interacting RNAs engaged in local (±10 kb flanking their genes), *cis* (in the same chromosome except local), and *trans* (in other chromosomes) interactions. Colors of dots represent different types of RNAs and sizes represent chromatin interaction levels. Labeled are representative caRNAs.

**
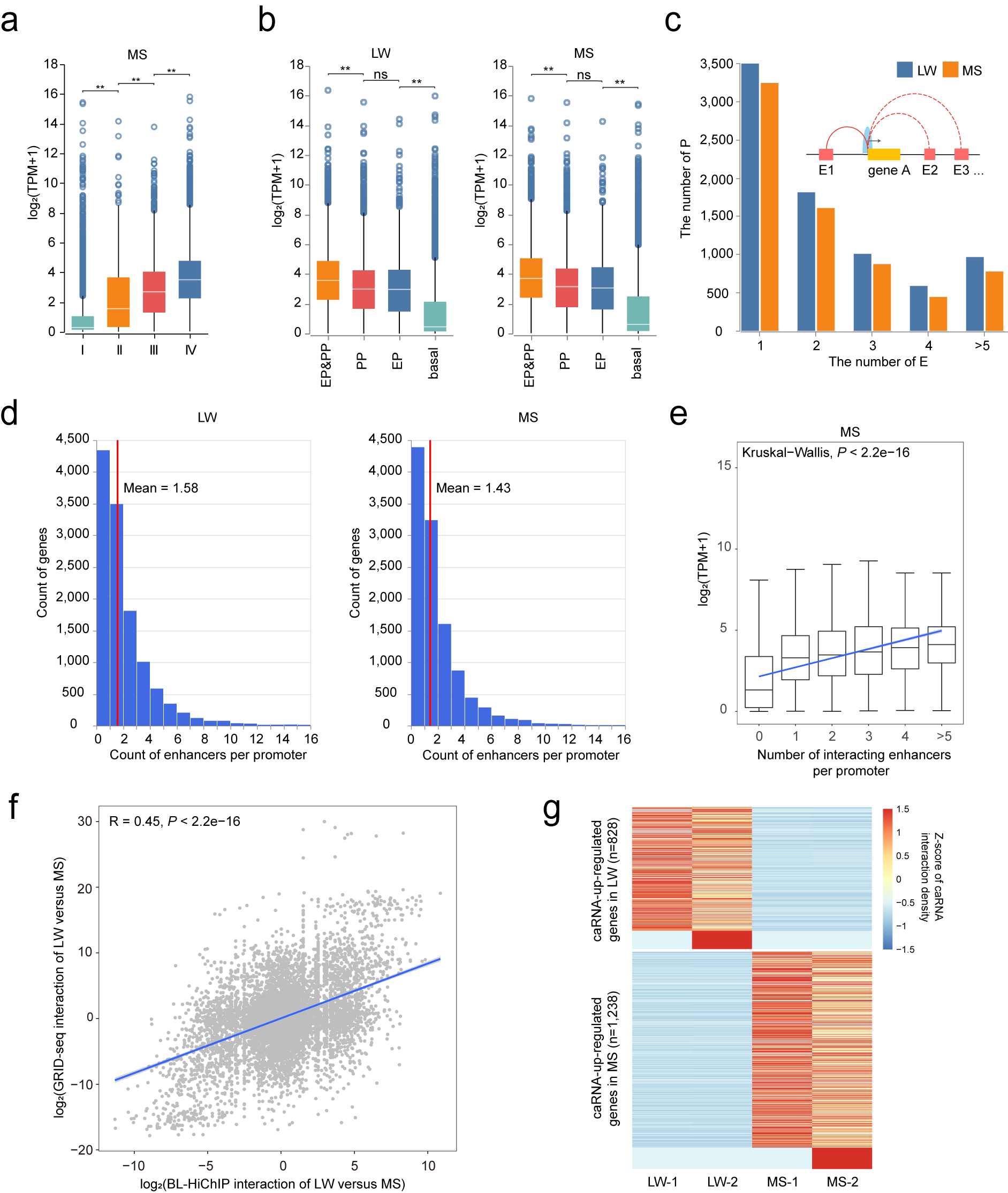
**

**Figure S5.** Chromatin loops affecting transcription regulation. (**a**) Expression of four types of genes in MS, type I (n=6,393), type II (n=1,010), type III (n=2,819), type IV (n=7,944) by Mann-Whitney-U single-tailed test with *P*-values from left to right: 5.2×10^-97^, 7.3×10^-27^, and 9.8×10^-77^. (**b**) LW expression analysis of genes involved in E-P and P-P interaction (n=4,375), only P-P interaction (n=3,557), only E-P interaction (n=1,690), basal genes (n=8,544). Basal genes: genes with no *cis*-regulatory elements detected. *P*-values from left to right: 1.2×10^-38^, 0.155, 5.5×10^-252^, by Mann-Whitney-U single-tailed test. MS expression analysis of genes involved in E-P and P-P interaction (n=3,848), only P-P interaction (n=3,632), only E-P interaction (n=1,474), basal genes (n=9,212). *P*-values from left to right: 1.7×10^-36^, 0.185, 2.2×10^-212^, by Mann-Whitney-U single-tailed test. (**c**) Summary bar chart of the number of P interacting with various numbers of E in LW and MS. (**d**) Histograms of the number of enhancers interacting with each promoter in LW and MS. Means are indicated. (**e**) Distribution of gene expression values grouped according to the numbers of interacting enhancers. Linear regression was performed on the mean gene expression values (*P*-value < 2.2 × 10^-16^, Kruskal−Wallis test for linear regression). (**f**) Scatter plot showing that the differences in chromatin-chromatin interaction and RNA-chromatin interaction levels were positively correlated between LW and MS (Pearson correlation, n = 9,598 promoters). The trend line from linear regression is shown. (**g**) Genes regulated by differential caRNA-chromatin interaction in LW (n=828) and MS (n=1,238).

**
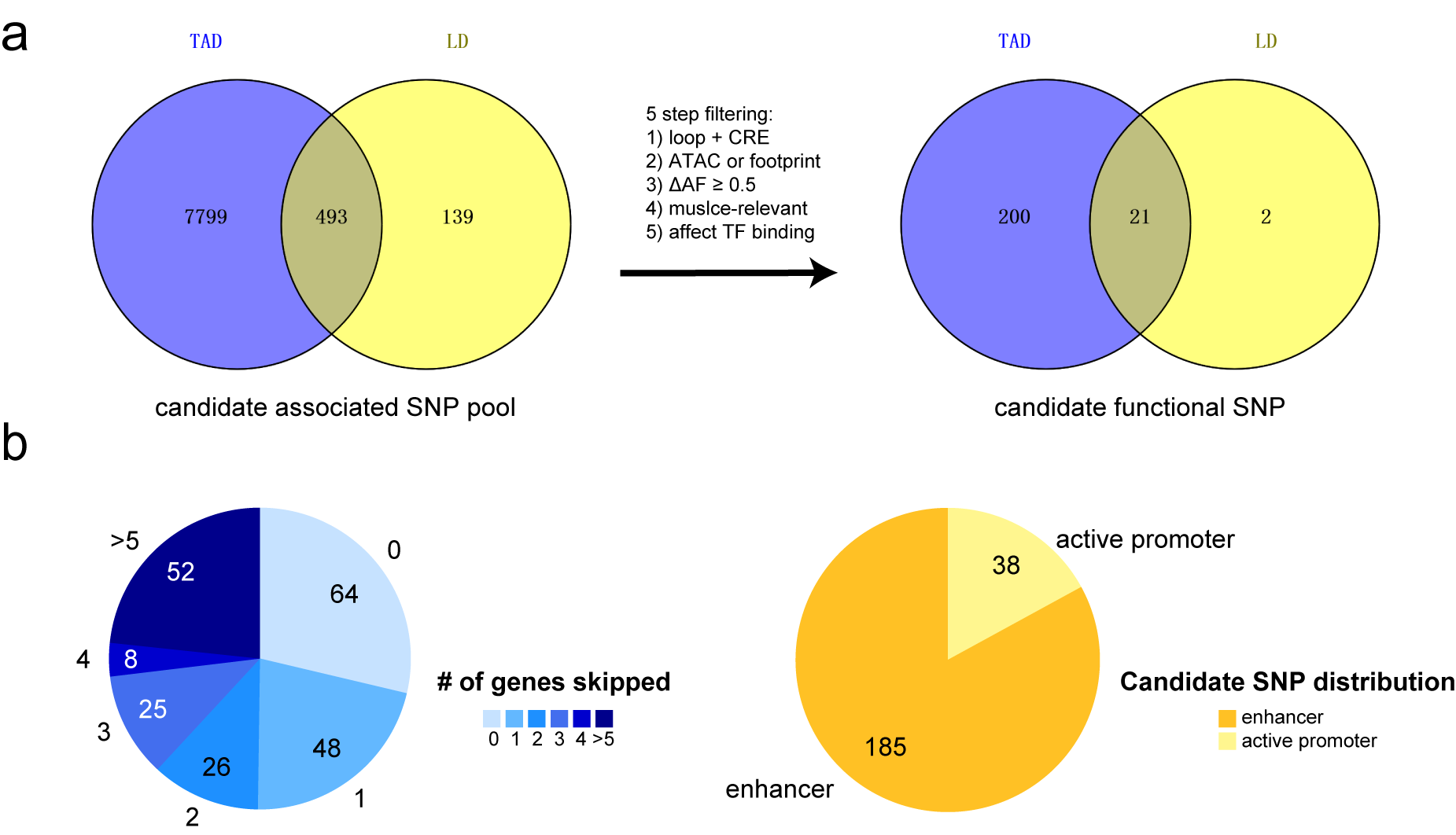
**

**Figure S6.** Comparison between TAD and LD approaches and statistics of newly-identified 223 SNPs. (**a**) For array-based GWAS, venn plot showing the comparison before and after multi-omics filtering. At last, TAD approach identified 221 candidate functional SNPs while LD approach identified 23 candidate functional SNPs, only 2 SNPs were exclusively identified by LD. (**b**) Pie charts showing the number of genes skipped for each candidate SNP-target gene interaction and the proportion of candidate functional SNPs residing on enhancers and active promoters.


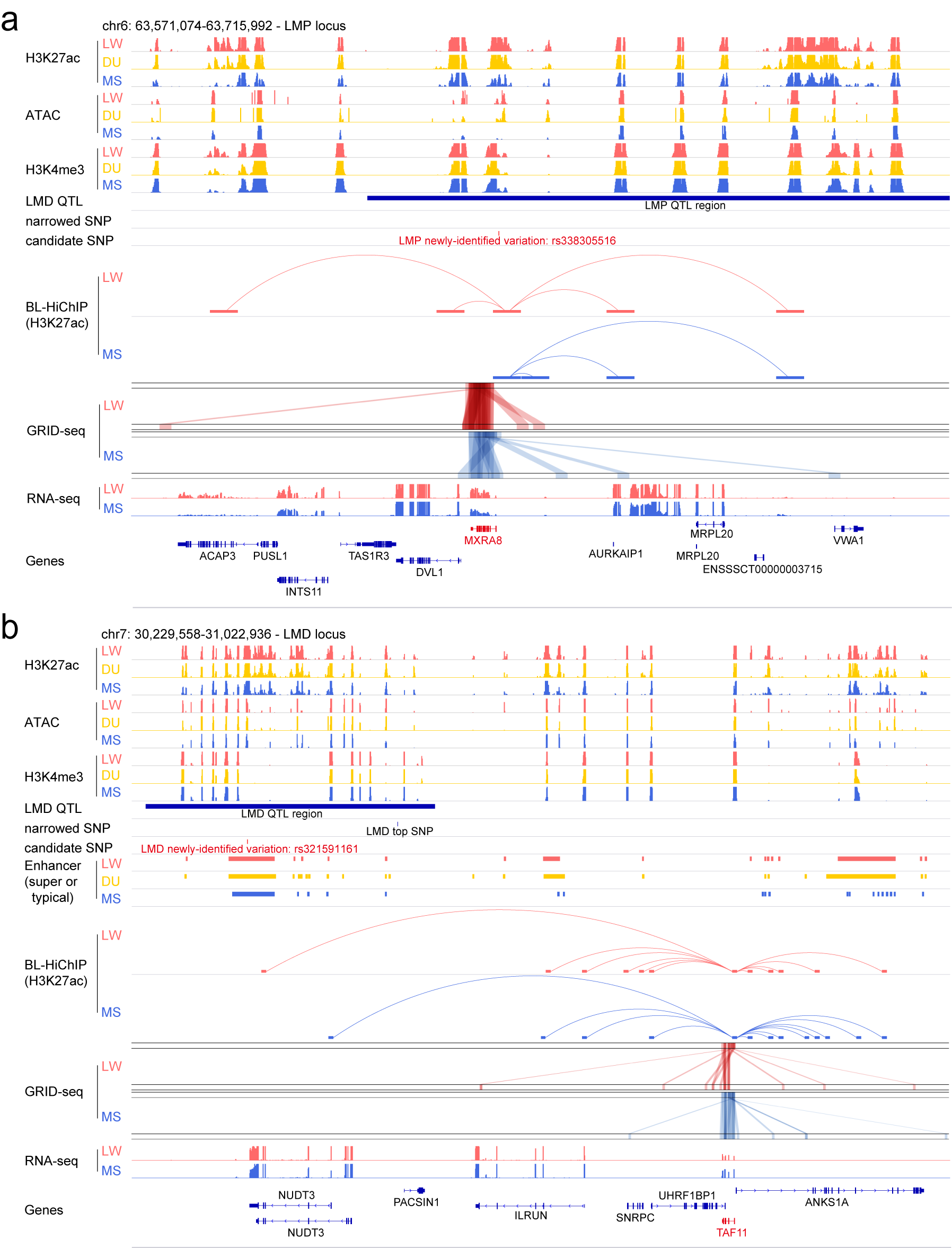


**Figure S7.** IGV plot of two candidate functional SNPs associated with LMP and LMD traits. (**a**) Examples of LMP QTL region in previous study, and newly-identified candidate functional SNP through multi-omics analyses. (**b**) Examples of LMD QTL region and top SNP in previous study, and newly-identified candidate functional SNP through multi-omics analyses.


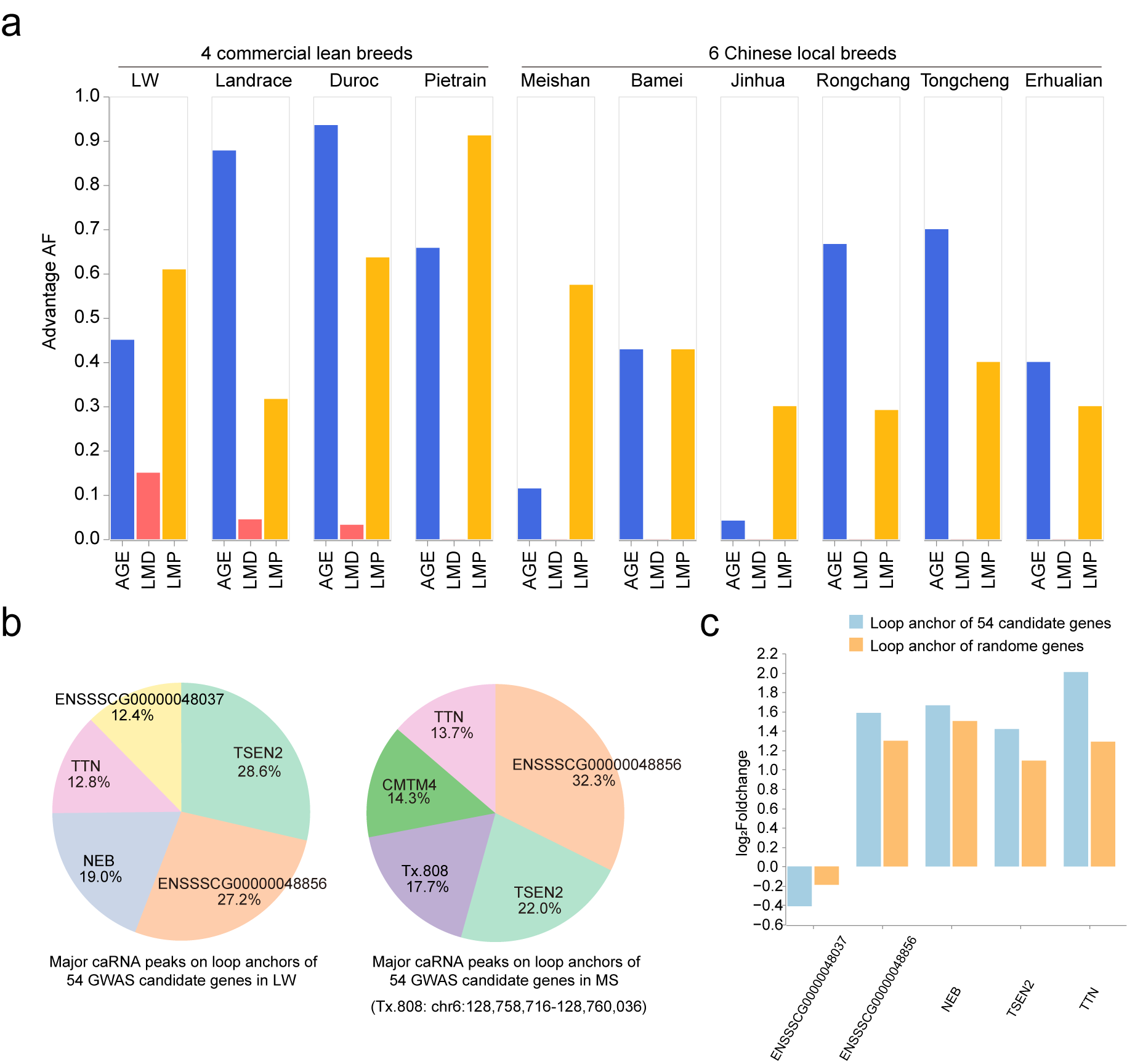


**Figure S8.** Allele frequency of three SNPs and major caRNAs associated with 54 GWAS target genes. (**a**) Frequency of target alleles with the enhanced phenotypic performance for AGE, LMP and LMD traits in each breed. (**b**) Major caRNAs that bound to loop anchors associated with 54 GWAS genes in LW and MS according to the count of caRNA peaks. (**c**) Bar plot showing enrichment of several major caRNAs on 54 GWAS genes, *TTN* was the most enriched compared with other caRNAs.
